# Breastfeeding and Later‐Life Cardiometabolic Health in Women With and Without Hypertensive Disorders of Pregnancy

**DOI:** 10.1161/JAHA.122.026696

**Published:** 2023-02-27

**Authors:** Maria C. Magnus, McKenzie K. Wallace, Jill R. Demirci, Janet M. Catov, Mandy J. Schmella, Abigail Fraser

**Affiliations:** ^1^ Center for Fertility and Health Norwegian Institute of Public Health Oslo Norway; ^2^ MRC Integrative Epidemiology Unit at the University of Bristol Bristol United Kingdom; ^3^ Population Health Sciences Bristol Medical School Bristol United Kingdom; ^4^ Martha S. Pitzer Center for Women, Children and Youth, College of Nursing The Ohio State University Columbus OH; ^5^ School of Nursing University of Pittsburgh Pittsburgh PA; ^6^ Department of Epidemiology University of Pittsburgh Pittsburgh PA; ^7^ Department of Obstetrics, Gynecology & Reproductive Science University of Pittsburgh Pittsburgh PA

**Keywords:** breastfeeding, cardiometabolic health, cardiovascular disease, hypertensive disorders of pregnancy, preeclampsia, Cardiovascular Disease, Lifestyle, Pregnancy

## Abstract

**Background:**

Breastfeeding is associated with improved cardiometabolic profiles decades after pregnancy. Whether this association exists for women who experience hypertensive disorders of pregnancy (HDP) is unknown. The authors examined whether breastfeeding duration or exclusivity are associated with long‐term cardiometabolic health, and whether this relationship differs by HDP status.

**Methods and Results:**

Participants (N=3598) were from the UK ALSPAC (Avon Longitudinal Study of Parents and Children) cohort. HDP status was assessed by medical record review. Breastfeeding behaviors were assessed by contemporaneous questionnaires. Breastfeeding duration was categorized as never, <1, 1 to <3, 3 to <6, 6 to <9, and 9+ months. Breastfeeding exclusivity was categorized as never, <1, 1 to <3, and 3 to 6 months. Measures of cardiometabolic health (body mass index, waist circumference, C‐reactive protein, insulin, proinsulin, glucose, lipids, blood pressure, mean arterial pressure, carotid intima‐media thickness, and arterial distensibility) were measured 18 years after pregnancy. Analyses were conducted using linear regression adjusting for relevant covariates. Breastfeeding was associated with improved cardiometabolic health (lower body mass index, waist circumference, C‐reactive protein, triglycerides, insulin, and proinsulin) in all women, but not for every breastfeeding duration. Interaction tests revealed additional benefits in women with a history of HDP, with the strongest benefit observed in the 6‐ to 9‐month breastfeeding category (diastolic blood pressure, −4.87 mm Hg [95% CI, −7.86 to −1.88], mean arterial pressure −4.61 [95% CI, −7.45 to −1.77], and low‐density lipoprotein cholesterol, −0.40 mmol/L [95% CI, −0.62 to −0.17 mmol/L]). Differences in C‐reactive protein and low‐density lipoprotein “survived” Bonferroni correction (*P*<0.001). Similar results were observed in the exclusive breastfeeding analyses.

**Conclusions:**

Breastfeeding may be a mechanism to reduce the cardiovascular disease sequela associated with HDP; however, there is a need to establish whether associations reflect a causal effect.

**Nonstandard Abbreviations and Acronyms**
ALSPAC: Avon Longitudinal Study of Parents and Children
SBP: systolic blood pressure
WC: waist circumference

**Clinical Perspective**

**What Is New?**

- Any breastfeeding is associated with improved cardiometabolic outcomes in women with and without a history of hypertensive disorders of pregnancy 18 years after pregnancy.
- Any breastfeeding is associated with greater blood pressure and cholesterol benefits for women with a history of hypertensive disorders of pregnancy 18 years after pregnancy.

**What Are the Clinical Implications?**

- Women experiencing a hypertensive disorder of pregnancy should receive support and encouragement to breastfeed to potentially reduce long‐term cardiovascular disease risk.

Cardiovascular disease (CVD) is the leading cause of death for women across the globe.^1^, ^2^, ^3^ Women who experience hypertensive disorders of pregnancy (HDP) have an increased risk of CVD‐related morbidity and mortality.^4^ HDP, including preeclampsia, superimposed preeclampsia, and gestational hypertension, complicate up to 15% of pregnancies.^5^ Women who experience HDP have a 2‐ to 3‐fold higher lifetime risk for development of and death from hypertension, stroke, and other CVDs compared with women who are normotensive during pregnancy.^4^, ^6^, ^7^, ^8^ Despite the known excess risk of CVD associated with HDP, effective interventions to mitigate the increased CVD risk in women with a history of HDP are lacking.

Breastfeeding is associated with both short‐ and long‐term cardiometabolic health benefits in women with uncomplicated pregnancies. In women with uncomplicated pregnancies, those who breastfed longer than 3 months had more favorable cholesterol, glucose, and insulin resistance profiles at 1 year postpartum compared with those who did not breastfeed or breastfed for <3 months.^9^, ^10^, ^11^ There is also evidence from cohort studies that breastfeeding for at least 6 months is associated with lower blood pressure (BP), appropriate‐range lipid profiles, and lower rates of type 2 diabetes, metabolic syndrome, and aortic and coronary calcification decades after pregnancy.^12^, ^13^, ^14^, ^15^

Studies that have investigated the long‐term associations between breastfeeding and maternal cardiometabolic health have been primarily conducted in the context of uncomplicated pregnancies. One previous study with long‐term follow‐up found that some breastfeeding compared with no breastfeeding was associated with a beneficial effect (lower diastolic BP [DBP] and lower carotid intima‐media thickness) for women with a history of HDP, but not to the same extent observed in women with uncomplicated pregnancies.^16^ Given the sparse evidence investigating the associations between breastfeeding and cardiometabolic health in women with a history of HDP, the objective of this study was to examine whether associations between breastfeeding intensity (duration and exclusivity) and cardiometabolic health varied according to the presence/absence of HDP.

## METHODS

Requests to access the data set may be submitted to the ALSPAC executive committee at the University of Bristol, see https://proposals.epi.bristol.ac.uk.

### Avon Longitudinal Study of Parents and Children

We studied women participating in ALSPAC (Avon Longitudinal Study of Parents and Children). ALSPAC is a prospective population‐based pregnancy cohort study that recruited 13 761 women residing in the former Avon Health Authority in England who gave birth to 14 541 children between April 1991 and December 1992.^17^ Detailed information on ALSPAC is available at (http://www.bristol.ac.uk/alspac). Ethical approval for this analysis was obtained from the ALSPAC Ethics and Law Committee and the Local Research Ethics Committees. ALSPAC participants provided informed consent before ALSPAC participation. Of the 13 606 women with singleton pregnancies participating in the cohort, 4423 completed a clinical follow‐up examination (≈18 years after pregnancy) between December 2008 and July 2011. Of the women who completed this follow‐up examination, 3659 were normotensive before pregnancy and had available data regarding breastfeeding and hypertensive disorders during the index pregnancy. Of the 3659 eligible women, 3598 reported consistent information about their breastfeeding history (never versus any) across questionnaires administered at 6 months postpartum and 15 months postpartum and were included in our study population (Figure). The index pregnancy is the pregnancy that occurred during ALSPAC participation between April 1991 and December 1992.

**Figure:**
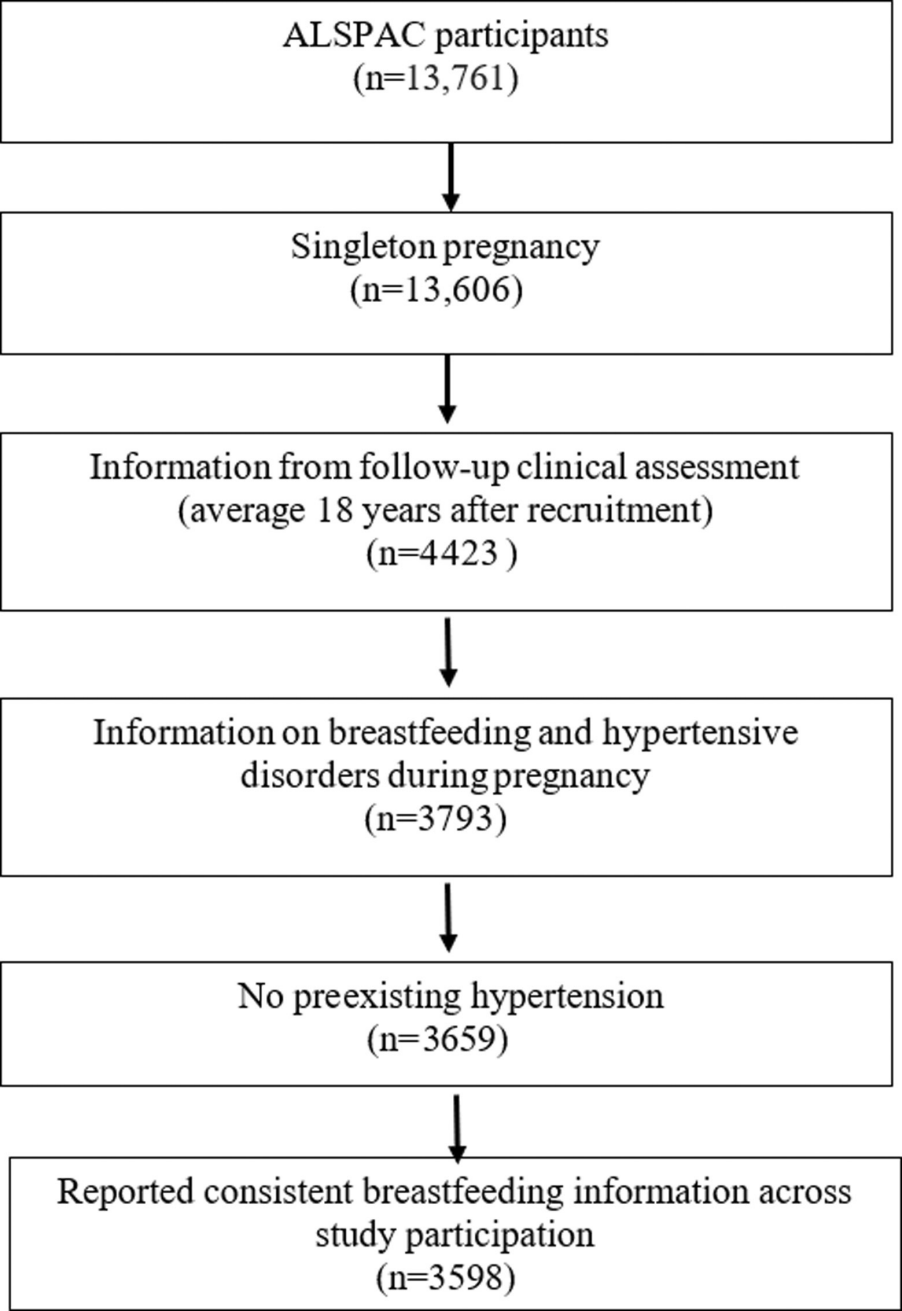
Flow diagram of the study population and analytic sample. ALSPAC indicates Avon Longitudinal Study of Parents and Children.

### Breastfeeding Duration

Participants completed questionnaires about breastfeeding behaviors at 6 months and 15 months postpartum. At 6 months postpartum, participants were asked, “Did you breastfeed?” Participant response options were “yes, I am still breastfeeding,” “yes, I breastfed but have now stopped,” and “I never breastfed.” If participants answered that they had stopped breastfeeding, they reported the infant's age in months at which they had stopped breastfeeding. At 15 months postpartum, participants were asked the same question. Duration of any breastfeeding was defined as the infant's age, in months, at which breastfeeding was stopped. We categorized the duration of any breastfeeding as never, <1 month, 1 to <3 months, 3 to <6 months, 6 to <9 months, and 9+ months. At the 6‐month questionnaire, participants were also asked, “Has your baby ever had the following: bottle of ordinary baby milk, powdered follow‐on milk, soy milk, goat's milk, hypoallergenic milk, cow's milk?” For each type of milk, participants reported the age that the infant received the milk source. Duration of exclusive breastfeeding was defined as the number of months the participant reported breastfeeding before the introduction of infant formula or other milk as assessed on the 6‐month questionnaire. We defined duration of exclusive breastfeeding as never breastfed, <1, 1 to <3, and 3 to 6 months. Never breastfeeding was the same group of participants for the any and exclusive analyses, and refers to participants who never breastfed at any point in the postpartum period of the index pregnancy.

### Hypertensive Disorders During Pregnancy

Preexisting hypertension (before pregnancy) was obtained by self‐report at recruitment (≈12 weeks gestation). HDP diagnoses (gestational hypertension or preeclampsia) were derived from BP and proteinuria measurements within the antenatal health record and defined according to the International Society for the Study of Hypertension in Pregnancy 2001 guidelines.^18^ Participants without preexisting hypertension were classified as having gestational hypertension if they had a systolic BP (SBP) ≥140 mm Hg and/or a DBP ≥90 mm Hg on at least 2 occasions first occurring after 20 gestational weeks. Preeclampsia was defined as gestational hypertension in combination with proteinuria (≥0.3 g/d).

### Cardiometabolic Health

Approximately 18 years after the index pregnancy, participants took part in a clinical examination conducted by trained study nurses, which included measurements of cardiometabolic health outcomes. Details about the collection of cardiometabolic health outcomes have been previously described in detail.^8^, ^17^, ^19^ Briefly, weight, height (used to calculate body mass index [BMI]), and waist circumference (WC) were measured with participants wearing light clothing and without shoes. BP was measured while participants were lying down using an Omron M6 monitor (Omron Healthcare UK Ltd). Two readings of SBP and DBP were recorded on each arm, and the mean of these 4 readings was used in analyses. Mean arterial pressure (MAP) was calculated as MAP=DBP+(SBP–DBP)/3. Fasting blood samples were obtained, centrifuged, separated, and frozen at −80 °C within 30 minutes. Plasma insulin and proinsulin were measured by enzyme‐linked immunosorbent assays (Mercodia). Plasma glucose was measured by automated enzymatic (hexokinase) method. Lipids were measured by automated analyzer with enzymatic methods. CRP (C‐reactive protein) was measured by automated particle‐enhanced immunoturbidimetric assay (Roche UK). Left and right common carotid artery measurements were assessed by ultrasound. Carotid intima‐media thickness and arterial distensibility were calculated as previously described.^19^

### Covariates

Participant race (White, Black, other), marital status (yes/no), manual social class (yes=partly skilled or unskilled occupation; no=professional, managerial/technical, or skilled occupation), university level education (yes/no), prepregnancy BMI (calculated from height and weight) (continuous), gravidity (primigravida; yes/no), and smoking at recruitment (yes/no) were assessed by questionnaires administered to participants during the index pregnancy. For the index pregnancy, information on maternal age at infant's birth (continuous), preterm birth status (gestational age <37 completed weeks), and presence of gestational diabetes (yes/no) were obtained from obstetric records. At the clinical examination (≈18 years after the index pregnancy), self‐reported information on the use of antihypertensives, cholesterol‐lowering agents, and diabetes medications was collected.

### Statistical Analysis

All analyses were conducted using Stata version 15 (StataCorp, LLC). χ^2^ tests were used to assess for differences in participant demographics and clinical characteristics, as well as for differences in length of breastfeeding by HDP status. We used linear regression to estimate differences in the measures of cardiometabolic health according to the duration of any and exclusive breastfeeding. We natural log transformed glucose, insulin, proinsulin, and CRP for all analyses. For these outcomes, we show the percent change in the outcomes according to the duration of breastfeeding. Other outcomes were evaluated on their original scale. Multivariable analyses were adjusted for maternal age at delivery, race, prepregnancy BMI, parity, smoking, marital status, manual occupational social class, and university education. BP outcomes were further adjusted for the use of antihypertensive medications. Glucose, insulin, and proinsulin were adjusted for the use of diabetes medications. Low‐density lipoprotein cholesterol (LDL‐C) and high‐density lipoprotein cholesterol (HDL‐C) were also adjusted for cholesterol‐lowering agents.

We tested for interactions between HDP and duration of any and exclusive breastfeeding by duration category on cardiometabolic health outcomes by including product terms in our regression analysis. We present stratified associations according to the presence of HDP for outcomes for which there was evidence of an interaction (*P*<0.05). We also conducted a sensitivity analysis excluding any participants who had a preterm delivery to assess whether differences in cardiometabolic outcomes in the HDP group were related to severity of the HDP, for which preterm birth is a proxy. Additionally, we used inverse probability weighting to examine the potential role of selection bias attributable to the participation rate at the follow‐up examination.^20^ We estimated the probability of attending the follow‐up visit 18 years after recruitment according to age, parity, race, marital status, social class, educational level, self‐reported prepregnancy BMI, smoking status, HDP, and preterm birth in the index pregnancy, and used these to weight analyses. Results with 95% CIs are reported. To account for multiple testing, we applied a Bonferroni correction (adjusted level of significance=0.001), which was based on the following number of tests: 14 outcomes×2 exposures×2 interactions. In our results section, we indicate which estimates “survived” the more stringent *P* value threshold. Pairwise correlations for the cardiometabolic outcomes are presented in Table S1, for completion.

## RESULTS

Maternal characteristics at the index pregnancy stratified by duration of any breastfeeding category are reported in Table 1. Participants who breastfed longer were older, more likely to be married, less likely to be of manual occupational social class, more likely to have a university education, had a lower self‐reported prepregnancy BMI, and were less likely to be primigravid, to smoke at recruitment, and to have developed HDP (Table 1). Those who attended the 18‐year clinical follow‐up versus those who did not attend were—at the time of recruitment during the index pregnancy—older, more likely to be married, less likely to be of a manual social class, more likely to have a university‐level education, have a lower prepregnancy BMI, less likely to smoke during pregnancy, have HDP, and have had a preterm birth (Table S2). Overall, participants who had HDP reported a shorter duration of both any and exclusive breastfeeding than those with normotensive pregnancies, with 17.6% of participants with HDP never breastfeeding compared with 13.6% for participants without HDP (Table 2).

**Table 1:** Demographic and Clinical Characteristics by Duration of Any Breastfeeding for the Index Pregnancy

Characteristics	Never (n=509)	<1 mo (n=317)	1 to <3 mo (n=428)	3 to <6 mo (n=653)	6 to <9 mo (n=651)	9+ mo (n=1040)	*P* value
Maternal age at birth of index child, mean (SD), y	28.2 (4.6)	28.7 (4.7)	28.7 (4.3)	29.7 (4.2)	30.1 (4.0)	31.3 (4.1)	<0.001
Race, n (%)							
White	496 (97.5)	308 (97.2)	418	629 (96.3)	626 (96.2)	1008 (96.9)	0.233
Black	<5	<5	(97.7)	5 (0.8)	9 (1.4)	8 (0.8)	
Other	<5	5 (1.6)	<5	10 (1.5)	7 (1.1)	17 (1.6)	
Missing	6 (1.2)	<5	<57 (1.6)	9 (1.4)	9 (1.4)	7 (0.7)	
Married, n (%)							
No	89 (17.5)	44 (13.9)	62 (14.5)	115 (17.6)	73 (11.2)	147 (14.1)	0.034
Yes	415 (81.5)	271 (85.5)	363 (84.8)	537 (82.2)	572 (87.9)	882 (84.8)	
Missing	5 (1.0)	<5	<5	<5	6 (0.9)	11 (1.1)	
Manual social class, n (%)							
No	337 (66.2)	232 (73.2)	313 (73.1)	484 (74.1)	529 (81.3)	774 (74.4)	0.001
Yes	120 (23.6)	50 (15.8)	75 (17.5)	99 (15.2)	67 (10.3)	147 (14.1)	
Missing	52 (10.2)	35 (11.0)	40 (9.4)	70 (10.7)	55 (8.5)	119 (11.4)	
University‐level education, n (%)							
No	485 (95.3)	292 (92.1)	376 (87.9)	542 (83.0)	464 (71.3)	698 (67.1)	0.001
Yes	18 (3.5)	20 (6.3)	46 (10.8)	106 (16.2)	182 (28.0)	335 (32.2)	
Missing	6 (1.2)	5 (1.6)	6 (1.4)	5 (0.8)	5 (0.8)	7 (0.7)	
Prepregnancy BMI, mean (SD), kg/m^2^	23.3 (4.0)	22.8 (3.6)	22.8 (3.6)	22.4 (3.3)	22.1 (2.9)	22.0 (2.9)	<0.001
Primigravid, n (%)							
No	348 (68.4)	179 (56.5)	258 (60.3)	378 (57.9)	395 (60.7)	734 (70.6)	<0.001
Yes	158 (31.0)	137 (43.22)	166 (38.8)	267 (41.0)	253 (38.9)	300 (28.9)	
Missing	<5	<5	<5	8 (1.2)	<5	6 (0.6)	
Smoking at recruitment, n (%)							
No	386 (75.8)	262 (82.7)	346 (80.8)	542 (83.0)	585 (89.9)	934 (89.8)	<0.001
Yes	121 (23.8)	54 (17.0)	78 (18.2)	103 (15.8)	61 (9.4)	93 (8.9)	
Missing	<5	<5	<5	8 (1.2)	5 (0.8)	13 (1.3)	
Pregnancy diabetes, n (%)							
None	486 (95.5)	303 (95.6)	411 (96.0)	626 (95.9)	630 (96.8)	1004 (96.5)	0.904
GD	5 (1.0)	<5	<5	<5	<5	5 (0.5)	
Pre‐GD	<5	<5	<5	<5	<5	<5	
Glycosuria	16 (3.1)	11 (3.47)	12 (2.8)	23 (3.5)	18 (2.8)	27 (2.6)	
HDP, n (%)							
None	416 (81.7)	270 (85.2)	350 (81.8)	569 (87.1)	551 (84.6)	913 (87.8)	0.001
Gestational hypertension	79 (15.5)	42 (13.3)	60 (14.0)	74 (11.3)	87 (13.4)	114 (11.0)	
Preeclampsia	14 (2.7)	5 (1.58)	18 (4.2)	10 (1.5)	13 (2.0)	13 (1.3)	
Preterm birth, n (%)							
No	486 (95.5)	296 (93.4)	415 (97.0)	629 (96.3)	621 (95.4)	1007 (96.8)	0.169
Yes	23 (4.5)	21 (6.6)	13 (3.0)	24 (3.7)	30 (4.6)	33 (3.2)	
Follow‐up measures							
Maternal age, mean (SD), y	46.6 (4.5)	46.9 (4.5)	47.1 (4.2)	48.0 (4.2)	48.4 (4.0)	49.6 (4.1)	<0.001
Medication usage, n (%)							
Antihypertensives	38 (7.47)	14 (4.42)	20 (4.67)	29 (4.44)	23 (3.53)	46 (4.42)	
Cholesterol‐lowering agents	10 (1.96)	<5	6 (1.40)	11 (1.68)	11 (1.69)	12 (1.15)	
Diabetes medications	10 (1.96)	<5	5 (1.17)	<5	6 (0.92)	12 (1.15)	

Categories with cell counts with <5 participants are designated as N<5 per Avon Longitudinal Study of Parents and Children policy to protect participant anonymity. BMI indicates body mass index; GD, gestational diabetes; and HDP, hypertensive disorders of pregnancy.

**Table 2:** Duration of Breastfeeding According to Presence of HDP

Breastfeeding outcome	Category	Total sample (N=3598)	No HDP (n=3069)	HDP (n=529)	*P* value
Any breastfeeding duration	Never	509 (14.2)	416 (13.6)	93 (17.6)	0.002
	<1 mo	317 (8.8)	270 (8.8)	47 (8.9)	
	1 to <3 mo	428 (11.9)	350 (11.4)	78 (14.7)	
	3 to <6 mo	653 (18.2)	569 (18.5)	84 (15.9)	
	6 to <9 mo	651 (18.1)	551 (18.0)	100 (18.9)	
	9+ mo	1040 (28.9)	913 (29.8)	127 (24.0)	
Exclusive breastfeeding duration	Never	509 (14.2)	416 (13.6)	93 (17.6)	0.006
	0 to <1 mo	871 (24.2)	734 (23.9)	137 (25.9)	
	1 to <3 mo	831 (23.1)	707 (23.8)	124 (23.3)	
	3–6 mo	1387 (38.6)	1212 (39.5)	175 (33.1)	

Values are expressed as number (percentage). HDP indicates hypertensive disorders of pregnancy.

### Relationship Between Duration of Any Breastfeeding and Cardiometabolic Outcomes at 18‐Year Follow‐Up

Results for outcomes for which there was no evidence of an interaction between HDP and duration of any breastfeeding are shown in Table 3 for the whole study sample (ie, regardless of HDP status). Before accounting for multiple testing, (ie, at the 5% significance level), duration of any breastfeeding was associated with lower BMI, WC, triglycerides, insulin, proinsulin, and higher HDL‐C after multivariable adjustment, but the evidence was weak, with 95% CIs crossing the null value for some breastfeeding categories. Duration of any breastfeeding in all categories was associated with lower CRP, with breastfeeding for 6 months, or longer, remaining significant after Bonferroni correction (*P*<0.001; Table 3). Breastfeeding for 6 to <9 months and 9+ months was consistently associated with more favorable cardiometabolic outcomes before accounting for multiple testing. Breastfeeding for 6 to <9 months was associated with a 0.55‐kg/m^2^ (95% CI, 0.15–0.96 kg/m^2^) lower BMI, 1.32‐cm (95% CI, 0.25–2.40 cm) smaller WC, 25.50% (95% CI, 11.97%–39.04%) lower CRP, 10.59% (95% CI, 3.40%–17.77%) lower insulin, 0.07‐mmol/L (95% CI, 0.03–0.12 mmol/L) higher HDL‐C, 0.07‐mmol/L (95% CI, 0.01–0.13) lower triglycerides, and 8.26% (95% CI, 1.69%–14.84%) lower proinsulin. However, after Bonferroni correction, the only surviving associations were between breastfeeding duration 6 to <9 month and CRP. Breastfeeding 9+ months was associated with 0.49‐kg/m^2^ (95% CI, 0.11–0.87 kg/m^2^) lower BMI, 1.25‐cm (95% CI, 0.25–2.26 cm) smaller WC, 20.93% (95% CI, 8.63%–33.23%) lower CRP, 0.08‐mmol/L (95% CI, 0.02–0.14 mmol/L) lower triglycerides, 6.77% (95% CI, 0.17%–13.37%) lower insulin, and 7.21% (95% CI, 1.06%–13.35%) lower proinsulin. Only the lower CRP levels remained significant after Bonferroni correction. There was no relationship between duration of breastfeeding and SBP, arterial distensibility, or carotid intima‐media thickness regardless of the *P* value threshold used (Table 3). Corresponding *P* values are available in Table S3.

**Table 3:** Adjusted Associations of Duration of Any Breastfeeding With Measures of Cardiometabolic Health

	<1 mo	1 to <3 mo	3 to <6 mo	6 to <9 mo	9+ mo
	Mean difference (95% CI)	Mean difference (95% CI)	Mean difference (95% CI)	Mean difference (95% CI)	Mean difference (95% CI)
BMI, kg/m^2^	−0.29 (−0.76 to 0.18)	−0.39 (−0.82 to 0.05)	−0.14 (−0.54 to 0.26)	−0.55 (−0.96 to −0.15)*	−0.49 (−0.87 to −0.11)*
WC, cm	−0.31 (−1.57 to 0.95)	−1.22 (−2.38 to −0.06)*	−0.55 (−1.61 to 0.51)	−1.32 (−2.40 to −0.25)*	−1.25 (−2.26 to −0.25)*
SBP, mm Hg	−0.50 (−2.16 to 1.16)	−1.31 (−2.84 to 0.22)	−0.01 (−1.40 to 1.38)	−0.35 (−1.77 to 1.07)	−1.02 (−2.35 to 0.30)
Arterial distensibility, mm	−0.00 (−0.02 to 0.01)	0.01 (−0.01 to 0.02)	−0.00 (−0.01 to 0.01)	0.00 (−0.01 to 0.02)	0.00 (−0.01 to 0.02)
CMIT, mm	−0.01 (−0.02 to 0.00)	−0.00 (−0.01 to 0.00)	0.00 (−0.01 to 0.01)	0.00 (−0.00 to 0.01)	−0.00 (−0.01 to 0.01)
CRP, % change	−17.12 (−32.76 to −1.47)*	−18.61 (−33.08 to −4.15)*	−19.87 (−32.89 to −6.84)*	−25.50 (−39.04 to −11.97)†	−20.93 (−33.23 to −8.63)†
HDL‐C, mmol/L	−0.01 (−0.07 to 0.04)	0.07 (0.02 to 0.12)*	0.02 (−0.02 to 0.07)	0.07 (0.03 to 0.12)*	0.04 (−0.00 to 0.08)
Triglycerides, mmol/L	−0.03 (−0.11 to −0.04)	−0.10 (−0.17 to −0.03)*	−0.02 (−0.08 to 0.04)	−0.07 (−0.13 to −0.01)*	−0.08 (−0.14 to −0.02)*
Insulin, % change	−1.44 (−9.77 to 6.89)	−9.77 (−17.69 to −1.84)*	−1.41 (−8.54 to 5.72)	−10.59 (−17.77 to −3.40)*	−6.77 (−13.37 to −0.17)
Proinsulin, % change	−1.19 (−9.11 to 6.37)	−5.88 (−13.17 to 1.41)	0.71 (−5.78 to 7.21)	−8.26 (−14.84 to −1.96)*	−7.21 (−13.35 to −1.06)*

Reference group=participants who never breastfed. All outcomes were adjusted for age at delivery, race, prepregnancy body mass index (BMI), parity, smoking, marital status, manual occupational social class, university education, and gestational diabetes. Blood pressure outcomes were further adjusted for use of antihypertensive medications. Glucose, insulin, and proinsulin outcomes were further adjusted for diabetes medications. High‐density lipoprotein cholesterol (HDL‐C) outcomes were further adjusted for cholesterol‐lowering medications. CMIT indicates carotid intima‐media thickness; CRP, C‐reactive protein; SBP, systolic blood pressure; and WC, waist circumference.

* *P*<0.05.

^†^ *P*<0.001 (Bonferroni correction).

Table 4 presents results stratified by HDP for outcomes for which there was evidence of an interaction between HDP and breastfeeding duration at *P*<0.05. Corresponding *P* values are available in Table S4. Interaction test *P* values are noted in Table S5. In women with HDP, DBP, MAP, LDL‐C, and glucose were lower among those who breastfed, although associations were not found for all breastfeeding duration categories. Among women diagnosed with an HDP, breastfeeding for 6 to <9 months was associated with the strongest benefit, with a 4.87‐mm Hg (95% CI, 1.88–7.86 mm Hg) lower DBP, 4.61 (95% CI, 1.77–7.45) lower MAP, and 0.40‐mmol/L (95% CI, 0.17–0.62 mmol/L) lower LDL‐C. After accounting for multiple comparisons, the finding related to LDL‐C remained statistically significant. Among women diagnosed with an HDP, breastfeeding for 1 to <3 months was associated with a 3.11 (95% CI, 0.17–6.04) lower MAP, 0.27‐mmol/L (95% CI, 0.04–0.50 mmol/L) lower LDL‐C, and 4.78% (95% CI, 0.59–8.98) lower glucose; however, only the difference in LDL‐C remained statistically significant after Bonferroni correction. In contrast, no associations between duration of breastfeeding and DBP, MAP, LDL‐C, and glucose were observed in women without HDP (Table 4). The distributions of the cardiometabolic outcomes according to duration of any breastfeeding are shown in Table S6. Tables S7 and S8 show *P* values from the test for a linear trend across breastfeeding categories. The unadjusted results are shown in Tables S9 and S10.

**Table 4:** Adjusted Associations of Duration of Any Breastfeeding With DBP, MAP, LDL‐C, and Glucose Stratified by Presence of HDP

	<1 month	1 to <3 months	3 to <6 months	6 to <9 months	9+ months
	Mean difference (95% CI)	Mean difference (95% CI)	Mean difference (95% CI)	Mean difference (95% CI)	Mean difference (95% CI)
No HDP					
DBP, mm Hg	−0.21 (−1.74 to 1.32)	−1.30 (−2.72 to 0.12)	−0.02 (−1.31 to 1.26)	−0.28 (−1.59 to 1.03)	−1.18 (−2.40 to 0.05)
MAP	−0.13 (−1.60 to 1.33)	−1.19 (−2.56 to 0.17)	0.25 (−0.97 to 1.48)	−0.07 (−1.33 to 1.19)	−0.95 (−2.12 to 0.23)
LDL‐C, mmol/L	0.03 (−0.10 to 0.16)	0.01 (−0.11 to 0.13)	0.01 (−0.10 to 0.12)	−0.05 (−0.16 to 0.06)	−0.05 (−0.15 to 0.05)
Glucose, % change	−0.27 (−1.92 to 1.38)	−0.77 (−2.36 to 0.82)	0.84 (−0.54 to 2.21)	−0.66 (−2.05 to 0.73)	−0.15 (−1.44 to 1.13)
HDP					
DBP, mm Hg	−3.25 (−6.85 to 0.34)	−3.08 (−6.17 to 0.02)	−2.67 (−5.76 to 0.41)	−4.87 (−7.87 to −1.88)*	−1.67 (−4.59 to 1.24)
MAP	−2.93 (−6.33 to 0.47)	−3.11 (−6.04 to −0.17)*	−2.87 (−5.79 to 0.04)	−4.61 (−7.45 to −1.77)*	−1.95 (−4.71 to 0.81)
LDL‐C, mmol/L	−0.26 (−0.53 to 0.02)	−0.41 (−0.66 to −0.17)†	−0.22 (−0.45 to 0.02)	−0.40 (−0.62 to −0.17)†	−0.27 (−0.50 to −0.04)*
Glucose, % change	−1.18 (−5.97 to 3.61)	−4.78 (−8.98 to −0.59)*	−1.91 (−6.18 to 2.36)	−3.98 (−8.05 to 0.09)	−2.96 (−6.95 to 1.02)

Reference group=participants who never breastfed. All outcomes were adjusted for age at delivery, race, prepregnancy body mass index, parity, smoking, marital status, manual occupational social class, university education, and gestational diabetes. Blood pressure outcomes were further adjusted for use of antihypertensive medications. Glucose, insulin, and proinsulin outcomes were further adjusted for diabetes medications. Low‐density lipoprotein cholesterol (LDL‐C) outcomes were further adjusted for cholesterol‐lowering medications. DBP indicates diastolic blood pressure; HDP, hypertensive disorders of pregnancy; and MAP, mean arterial pressure.

* *P*<0.05.

^†^ *P*<0.001 (Bonferroni correction).

### Relationship Between Duration of Exclusive Breastfeeding and Cardiometabolic Outcomes at 18 Years of Follow‐Up

Associations between duration of exclusive breastfeeding and cardiometabolic outcomes were similar to those observed for any breastfeeding and cardiometabolic outcomes. As seen with duration of any breastfeeding, all categories of exclusive breastfeeding compared with never breastfed were associated with lower concentrations of CRP; lower concentrations of CRP observed in the 1 to <3 months and 3 to <6 months categories for exclusive breastfeeding survived Bonferroni correction (Table S11). Corresponding *P* values are available in Table S12. Exclusive breastfeeding for 3 to 6 months compared with never breastfed was associated with a smaller WC and lower BMI, but these associations did not remain after Bonferroni correction. Exclusive breastfeeding for 1 month or longer was associated with improved HDL‐C, lower triglycerides, and lower insulin, but these differences did not remain statistically significant after Bonferroni correction. As seen with any breastfeeding, there was no evidence of associations between duration of exclusive breastfeeding and SBP, arterial distensibility, and carotid intima‐media thickness (Table S11).

For comparison, we show stratified results according to HDP for outcomes that showed evidence of an interaction with duration of any breastfeeding as described above (DBP, MAP, LDL‐C, and glucose), with interaction tests shown in Table S13. All exclusive breastfeeding categories were associated with lower LDL‐C, but the association remained statistically significant after Bonferroni correction only for patients who exclusively breastfed for 3 to <6 months (Table S14). Exclusive breastfeeding for 1 to <3 months was associated with lower DBP and lower blood glucose in women with HDP. Exclusive breastfeeding for 1 to <3 months and 3 to 6 months was associated with lower MAP in women with HDP. These differences did not remain statistically significant after accounting for multiple testing (Table S14). Corresponding *P* values are available in Table S15. Unadjusted results are presented in Tables S16 and S17.

### Sensitivity Analyses

Because severity of HDP may influence breastfeeding initiation and duration, we assessed whether breastfeeding rates differed for those with preterm HDP (a proxy for severity of HDP) and those with term HDP. The proportion of individuals with any breastfeeding for 3 months or less was similar between participants with HDP only and those with HDP and preterm birth (Table S18). The proportion of participants who never breastfed was slightly higher among participants with HDP only (17.8%) compared with those who experienced an HDP and preterm birth (14.7%). Sensitivity analyses excluding all preterm births largely indicated that the relationships between breastfeeding and cardiometabolic outcomes excluding all preterm births were similar to the results reported above that included all participants, indicating that preterm birth status did not contribute to our findings (results not shown). Inverse probability weighting analyses to assess for potential selection bias related to which individuals participated in the clinical follow‐up visit also yielded similar findings (Tables S18–S22). We also conducted a sensitivity analysis stratified with parity of 1 and >1. The results were largely similar, indicating that our findings are likely not attributable to residual confounding by parity (Tables S23–S30).

## DISCUSSION

Our results suggest that duration of any breastfeeding is associated with improved BMI, WC, CRP, HDL‐C, triglycerides, insulin, and proinsulin in women regardless of their history of HDP almost 2 decades after pregnancy. In women with a history of HDP, an additional benefit was observed—any breastfeeding duration was associated with DBP, MAP, LDL‐C, and glucose. The improved cardiometabolic benefits were most strongly observed in those who breastfed for 6 to <9 months, with associations for lower CRP and LDL‐C remaining statistically significant even at the Bonferroni‐corrected significance level. The additional benefit observed in women with a history of HDP is especially important given the increased risk for CVD, including hypertension, among women with a history of HDP.^4^ We observed similar relationships between the duration of exclusive breastfeeding and cardiometabolic health as was observed between any breastfeeding and cardiometabolic health.

We did not observe a dose‐dependent response between duration of any breastfeeding and cardiometabolic outcomes as we had expected. This could be attributable to misclassification of breastfeeding duration caused by recall bias or the fact that our characterization of duration did not capture breastfeeding intensity (eg, frequency and volume of breastfeeding) that may impact our outcomes of interest. For example, individuals who breastfeed a few times a day and individuals who provided the majority of their child's nutritional intake from breastmilk, who both reported breastfeeding cessation at 9 months, are treated the same in the analysis. Relatedly, this may explain why 6 to 9 months more strongly associated with cardiometabolic outcomes than breastfeeding for longer than 9 months. Breastfeeding is recommended as the main nutritional source until 6 months of age, but after 6 months, families introduce solid foods at varying rates.^21^ As time goes on, infants are likely to get more of their nutritional support from foods besides breastmilk, and breastfeeding frequency and duration of breastfeeding sessions may decline. Women in the longest breastfeeding duration category likely had a wide range in frequency of breastfeeding when breastfeeding for more than 9 months, which may explain in part why we did not see an additional benefit in the 9+ month cohort compared with the 6‐ to 9‐month cohort.

During pregnancy, a physiologic shift occurs that leads to increased blood volume, increased maternal fat stores, elevated lipids, and increased insulin resistance to support the growth and developmental needs of the fetus.^22^, ^23^ According to the metabolic reset hypothesis, breastfeeding triggers a substantial metabolic shift back to the prepregnancy state. The metabolic shift mobilizes pregnancy‐accumulated fat stores to support the caloric demands of milk production, resulting in improved lipid profiles, improved insulin resistance, and weight loss.^22^ If the metabolic shift of pregnancy is not reversed after birth, the physiologic atherogenic state of pregnancy persists and increases risk for cardiometabolic diseases such as hypertension, hypercholesteremia, and type 2 diabetes.^24^ It has also been hypothesized that oxytocin, a key hormone that regulates lactation, promotes vascular homeostasis that results in decreased SBP and DBP.^25^, ^26^ The alternative metabolic preset hypothesis postulates that prepregnancy cardiometabolic health factors impact the capacity for successful lactation/milk production and thus the initiation and duration of lactation, which may drive the observed association between breastfeeding and mothers' postpartum cardiometabolic health.^24^ Our findings that breastfeeding was associated with improved cardiometabolic outcomes after adjusting for prepregnancy BMI and cardiometabolic medication use support the metabolic reset hypothesis.

Future studies should also consider investigating other mechanisms that may contribute to relationships between breastfeeding and maternal cardiometabolic outcomes. For example, it is unclear whether there are differences in cardiometabolic outcomes among individuals who primarily breastfeed directly at breast (with potential for increased oxytocin surge with skin‐to‐skin contact) versus those who feed their pumped/expressed milk. Recent studies have also begun to investigate the inverse relationship between vasopressin and oxytocin in breastfeeding women, as vasopressin and oxytocin compete for receptors and have different effects.^27^, ^28^ However, the role of vasopressin in long‐term maternal cardiovascular health has not been investigated. This line of inquiry may be particularly relevant to women with a history of HDP given the associations between vasopressin and preeclampsia.^29^

Any breastfeeding was associated with lower MAP and DBP and LDL‐C for women with a history of HDP but not in women with normotensive pregnancies. Particularly, the group of women who were diagnosed with an HDP and who breastfed for 6 to <9 months had a nearly 5‐mm Hg lower DBP and MAP compared with the group who never breastfed, which is a clinically meaningful difference in BP. Specifically, meta‐analyses show the mean improvement in DBP from antihypertensive medications, the gold standard for treating hypertension, is 3 to 5 mm Hg for DBP.^30^, ^31^ Moreover, a reduction in diastolic BP of just 2 mm Hg is expected to reduce stroke risk by 14% and coronary heart disease by 6%, and is considered clinically significant.^32^, ^33^ The lack of association in women with uncomplicated pregnancies is incongruent with findings reported from previous studies of women with normotensive pregnancies, which have reported an association between breastfeeding and BP. However, previous studies assessed BP as a dichotomous outcome yes versus no hypertension,^12^, ^14^ whereas we assessed BP as a continuous outcome. Our findings agree with those reported by Countouris and colleagues, who also reported lower DBP in women with a history of HDP who breastfed compared with those who did not breastfeed.^16^, ^34^ Yu and colleagues also report improved cholesterol profiles in women with a history of pregnancy complications known to increase risk for cardiovascular disease, including HDP, but report improved HDL‐C and triglycerides, which we observed for all women who breastfeed, not just those with a history of HDP.^35^ However, Yu et al separated breastfeeding categories into none, <6 months, and more than 6 months, and also included numerous adverse outcomes, not just HDP. The lack of a relationship between duration of breastfeeding and BP in women who experienced a normotensive pregnancy may be partly because their pregnancy BP had less “opportunity” to improve postpartum. It is also plausible that women with a history of HDP are more sensitive to, or receive greater benefit from, the effects of oxytocin on BP than women who do not have a history of HDP, but this requires further investigation.

Our results show that breastfeeding may have cardiometabolic benefits for women, with possible additional benefits for women with HDP, suggesting the importance of breastfeeding for women with HDP. However, women with a history of HDP are known to have lower rates of breastfeeding.^36^ In our study, we observed a lower rate of breastfeeding initiation and continuation among women with HDP compared with women without HDP. These rates did not differ by preterm birth status, which we used as a proxy for severity of HDP. We have previously identified barriers to breastfeeding initiation and continuation in women with HDP, including maternal and infant separation, medical management (eg, magnesium sulfate and antihypertensive medications), maternal fluid imbalance (eg, third spacing), and delayed lactogenesis II.^37^ Despite these potential barriers to breastfeeding initiation and continuation in women with a history of HDP, Cordero et al (2012) showed that the strongest predictor of breastfeeding initiation in women with severe preeclampsia was the intention to breastfeed before delivery.^36^ As such, there is a critical need to develop interventions and health policies that address barriers to breastfeeding and encourage breastfeeding in this high‐risk population.

This analysis has several strengths. First, we had access to data regarding breastfeeding behaviors that were prospectively collected at multiple time points over the first 15 months postpartum. In other studies, documentation of breastfeeding intensity is often less granular (eg, breastfeeding: yes/no; breastfeeding <6 or ≥6 months), retrospectively collected (and subject to recall bias), and often does not include an assessment of exclusivity. This analysis also involved a large, well‐characterized cohort that included a large number of participants who experienced HDP. Previous studies that have investigated cardiometabolic outcomes by breastfeeding behavior have had fewer participants diagnosed with HDP, perhaps limiting the statistical power to detect some of the interaction effects we observed in our analysis (eg, 544 participants with HDP in our study versus 75 to 281 in previous studies)^16^, ^34^, ^36^, ^38^ or excluded participants with a history of HDP. Our study assessed the association of breastfeeding with cardiometabolic health assessed ≈18 years after recruitment into ALSPAC. Of the limited number of studies investigating this relationship in women who experience an HDP, many are limited to short‐term follow‐up within the first year following pregnancy. Last, we calculated a Bonferroni‐corrected *P* value threshold and interpreted results taking this corrected threshold into account to protect against type 1 errors. This may be overcautious given that some outcomes were moderately correlated; however, we provide the reader with *P* values for all outcomes to aid in their interpretation of the results.

This analysis also has limitations. These data reflect pregnancy care and breastfeeding practices in the United Kingdom in the 1990s and may not be similarly applicable today. For example, interpretations of exclusive and any breastfeeding may be different today with expanded access to and use of electric breast pumps and evolving guidance and conventions on type and timing of complementary food introduction. Additionally, lactation support and recommendations in the 1990s differed from today, which may influence breastfeeding behaviors or success. Second, the demographic characteristics of participants, such as self‐identified race and BMI, may not be generalizable to other countries, nor reflect the current demographic and clinical characteristics of society today (eg, increased prevalence of obesity). Third, data on breastfeeding behaviors and pregnancy outcomes for pregnancies other than the index pregnancy were not collected but may impact long‐term cardiometabolic outcomes. In particular, we do not have data regarding previous HDP, which influences cardiovascular outcomes. Fourth, despite robust metrics for quantifying breastfeeding exposure, there is still a risk of misclassification through participant misinterpretation of the breastfeeding assessment items or inaccurate recall of breastfeeding behavior. Relatedly, the way the breastfeeding questions were presented to participants did not allow participants to report whether they were using a breast pump. Fifth, we did not have data regarding medical diagnoses that could potentially preclude breastfeeding such as mastectomy. Finally, this was an observational study; therefore, confidence that associations reflect causal relationships is limited. Moreover, participants who attended the follow‐up visits, and therefore were included in the analysis were older, more likely to be of White race, married, have a higher socioeconomic status, be nulliparous, and more likely to have had a preterm birth. They also had a lower BMI, were less likely to smoke, and were less likely to have experienced an HDP. However, we used inverse probability weighting, sensitivity analyses, and adjustment strategies to reduce the risk of bias caused by selection, reverse causation, and confounding, but cannot rule out residual confounding.

## CONCLUSION

Our results contribute to the evidence demonstrating that women with HDPs experience a similar or slightly enhanced cardioprotective benefit associated with breastfeeding as women who experienced a normotensive pregnancy. Because women who experience an HDP have excess risk of poor cardiometabolic health in the years following pregnancy,^4^, ^5^ this high‐risk group may derive the greatest benefit from breastfeeding should our results reflect a causal effect. Future research should focus on replication of the evidence establishing an association between breastfeeding and cardiometabolic health, especially for women with HDP. Additionally, further research is needed to identify barriers to breastfeeding initiation and continuation, especially among women with HDP. Identifying barriers to breastfeeding initiation and continuation in women with HDP in particular, and tailoring interventions to support these women, could positively impact the cardiometabolic health of women decades after pregnancy and ultimately prevent morbidity and mortality related to CVD.

## Sources of Funding

The UK Medical Research Council, Wellcome (grant reference: 102215/2/13/2) and the University of Bristol provide core support for ALSPAC. A comprehensive list of grant funding is available on the ALSPAC website (http://www.bristol.ac.uk/alspac/external/documents/grant‐acknowledgements.pdf). This work was partly supported by the Research Council of Norway through its Centres of Excellence funding scheme, project number 262700. M.C.M. has received funding from the European Research Council under the European Union's Horizon 2020 research and innovation programme (grant agreement number 947684). This work was also supported by NIH/NINR T32NR009759.

## Disclosures

The authors have no disclosures to report.

## Supporting information

Tables S1–S30
