# Supplementary material for: Breastfeeding and Later‐Life Cardiometabolic Health in Women With and Without Hypertensive Disorders of Pregnancy
Source: J Am Heart Assoc. 2023 Feb 27;12(5):e026696. doi: 10.1161/JAHA.122.026696 (PMC10111449; doi:10.1161/JAHA.122.026696)
Supplement: Supplementary file 1 — Tables S1–S30 [file JAH3-12-e026696-s001.pdf]

SUPPLEMENTAL MATERIAL

Table S1. Pairwise correlations for cardiometabolic outcomes

	1	2	3	4	5	6	7	8	9	10	11	12	13	14
Triglycerides (1)	1.00													
HDL cholesterol (2)*	-0.44	1.00												
Body Mass Index (3)	0.33	-0.38	1.00											
Waist circumference (4)	0.40	-0.41	0.90	1.00										
Carotid intima-media thickness (5)	0.06	0.01	0.06	0.07	1.00									
Arterial distensibility (6)	-0.13	0.06	-0.05	-0.07	0.16	1.00								
Insulin (7)	0.38	-0.37	0.50	0.52	0.02	-0.11	1.00							
Proinsulin (8)	0.37	-0.28	0.47	0.49	0.03	-0.07	0.71	1.00						
Glucose (9)	0.25	-0.15	0.21	0.24	0.08	-0.06	0.40	0.42	1.00					
C-reactive protein (10)	0.28	-0.30	0.55	0.54	0.01	-0.05	0.33	0.29	0.13	1.00				
Systolic blood pressure (11)	0.20	-0.10	0.31	0.34	0.20	0.03	0.25	0.24	0.16	0.21	1.00			
LDL cholesterol (12) †	0.29	-0.22	0.17	0.20	0.08	-0.11	0.11	0.09	0.04	0.12	0.13	1.00		
Diastolic blood pressure (13)	0.17	-0.09	0.25	0.27	0.09	-0.14	0.23	0.21	0.09	0.19	0.66	0.11	1.00	
Mean arterial pressure (14)	0.20	-0.10	0.30	0.32	0.14	-0.08	0.26	0.25	0.13	0.22	0.86	0.13	0.95	1.00

* HDL- high density lipoprotein
† LDL- low density lipoprotein

Table S2. Distribution of background characteristics among eligible women who did and did not attend the clinical follow-up evaluation 18 years after recruitment

Characteristics	Did not attend clinical follow-up (n=9,947)	Attended clinical follow-up (n=3,659)	p-value
Maternal age at birth of index child, y, mean (SD)	27.3 (5.0)	29.8 (4.4)	<0.001
Race, n (%)			0.001
White	7,785 (97.0)	3,546 (98.0)	
Black	101 (1.3)	26 (0.7)	
Other	140 (1.7)	46 (1.3)	
Missing	1,921	41	
Married, n (%)			<0.001
No	2,436 (27.1)	540 (14.9)	
Yes	6,541 (72.9)	3,091 (85.1)	
Missing	970	28	
Manual social class, n (%)			<0.001
No	5,054 (70.2)	2,713 (82.6)	
Yes	2,146 (29.8)	572 (17.4)	
Missing	2,747	374	
University level education, n (%)			<0.001
No	7,315 (90.2)	2,911 (80.3)	
Yes	799 (9.9)	714 (19.7)	
Missing	1,833	34	
Pre-pregnancy BMI, kg/m^2, mean (SD)	23.1 (23.1)	22.5 (3.3)	<0.001
Nulliparous, n (%)			0.002
No	5,876 (66.9)	2,323 (63.9)	
Yes	2,913 (33.1)	1,310 (36.1)	
Missing	1,158	26	
Smoking during pregnancy, n (%)			<0.001
No	6,045 (69.0)	3,105 (85.7)	
Yes	2,721 (31.04)	520 (14.3)	
Missing	1,181	34	
Pregnancy diabetes mellitus, n (%)			0.624
None	7,697 (95.8)	3,519 (96.2)	
GDM	36 (0.5)	18 (0.5)	
Pregestational diabetes mellitus	28 (0.4)	14 (0.4)	
Glycosuria	273 (3.4)	108 (3.0)	
Missing	1,913	0	
Hypertensive disorders of pregnancy, n (%)			<0.001
None	6,067 (82.3)	3,115 (85.1)	
Gestational hypertension	1,142 (15.5)	470 (12.9)	
Preeclampsia	165 (2.2)	74 (2.0)	
Missing	2,141	0	
Preterm birth, n (%)			<0.001
No	9,058 (91.4)	3,514 (96.0)	
Yes	858 (8.7)	145 (4.0)	
Missing	31	0	

Table S3. P values for adjusted associations of duration of any breastfeeding with measures of cardiometabolic health

	< 1 month	1-<3 months	3-<6 months	6-<9 months	9+ months
Body Mass Index (kg/m2)	.23	.08	.48	.007	.01
Waist Circumference (cm)	.63	.04	.31	.02	.02
Systolic BP (mmHg)	.56	.09	.98	.63	.13
Arterial distensibility (mm)	.82	.48	.95	.53	.68
cIMT(mm) *	.05	.46	.61	.36	.80
CRP (% change) †	.03	.01	.003	.000	.001
HDL (mmol/L) ‡	.61	.007	.34	.002	.05
Triglycerides (mmol/L)	.40	.006	.48	.03	.006
Insulin (% change)	.74	.02	.70	.004	.04
Proinsulin (% change)	.77	.11	.83	.01	.02

* cMIT - carotid intima-media thickness
† CRP- C- reactive protein
‡ HDL- high density lipoprotein cholesterol

Table S4. P values for adjusted associations of duration of any breastfeeding with diastolic blood pressure, mean arterial pressure, LDL-cholesterol, and glucose stratified by presense of hypertesive disorders of pregnancy (HDP)

	< 1 month	1-<3 months	3-<6 months	6-<9 months	9+ months
No Hypertensive disorders of pregnancy					
Diastolic BP (mmHg)	.78	.07	.97	.67	.06
Mean arterial pressure	.86	.09	.69	.92	.11
LDL cholesterol (mmol/L)*	.68	.86	.82	.38	.32
Glucose (% change)	.75	.34	.23	.35	.81
Hypertensive disorder of pregnancy					
Diastolic BP (mmHg)	.08	.05	.09	.002	.26
Mean arterial pressure	.09	.04	.05	.002	.17
LDL cholesterol (mmol/L)*	.07	.001	.07	.001	.02
Glucose (% change)	.63	.03	.38	.06	.14

*LDL- low density lipoprotein

Table S5. P-values from the Interaction Test of Any Breastfeeding and Hypertensive Disorders of Pregnancy on Cardiometabolic Health Measurements

Outcome	< 1 month	1-<3 months	3- <6 months	6- <9 months	9 months
Body-mass index	0.103	0.563	0.117	0.585	0.750
Waist circumference	0.195	0.867	0.278	0.398	0.573
Systolic blood pressure	0.460	0.376	0.093	0.125	0.946
Diastolic blood pressure	0.125	0.421	0.178	0.023	0.642
Mean arterial pressure	0.173	0.360	0.107	0.028	0.768
cIMT*	0.912	0.050	0.306	0.898	0.485
Arterial distensibility	0.371	0.961	0.322	0.787	0.897
Glucose	0.782	0.053	0.158	0.047	0.078
Insulin	0.992	0.678	0.398	0.804	0.782
Proinsulin	0.446	0.823	0.849	0.742	0.386
Trigylcerides	0.358	0.245	0.803	0.376	0.850
HDL-cholesterol †	0.605	0.295	0.987	0.326	0.923
LDL-cholesterol ‡	0.139	0.004	0.201	0.020	0.202
C-reactive protein	0.662	0.656	0.851	0.951	0.995

*cMIT - carotid intima-media thickness

† HDL- high density lipoprotein

‡ LDL- low density lipoprotein

p <.05 indicates a significant interaction test between any breastfeeding by duration category and hypertensive disorder of pregnancy

Table S6. Distribution of cardiometabolic health measurements at 18 year follow-up according to duration of any breastfeeding for index pregnancy

	Duration of Any Breastfeeding (months)						
Characteristics	Never (n=509)	<1 (n=317)	1-<3 (n=428)	3-<6 (n=653)	6-<9 (n=651)	9+ (n=1,040)	p-value*
BMI, kg/m^2, mean (SD)	27.95 (6.08)	26.96 (5.36)	26.85 (5.46)	26.53 (5.20)	25.72 (4.68)	25.65 (4.60)	<0.001
Waist circumference, cm, mean (SD)	87.39 (13.87)	85.53 (12.63)	84.62 (12.82)	84.25 (11.97)	82.60 (11.31)	82.57 (10.79)	<0.001
SBP, mmHg, mean (SD)†	118.99 (12.34)	117.8 (11.62)	116.9 (12.40)	118.2 (12.29)	117.7 (11.82)	117.3 (12.52)	0.066
DBP, mmHg, mean (SD)‡	73.82 (10.64)	72.52 (10.57)	71.74 (9.56)	72.56 (9.23)	72.03 (10.70)	71.69 (10.49)	0.004
cIMT, mm, mean (SD) §	0.554 (0.061)	.546 (.056)	0.552 (0.065)	0.560 (0 063)	0.562 (0.060)	0 .562 (0 .064)	<0.001
Arterial distensibility, mm, mean (SD)	0.505 (.119)	0.501 (.108)	0 .509 (0 .120)	0.497 (0.122)	0.499 (0.115)	0 .491 (0 115)	0.044
Glucose, mmol/L, mean (SD)	5.39 (1.27)	5.29 (1.01)	5.24 (1.03)	5.29 (0.78)	5.19 (0.54)	5.27 (1.02)	0.014
Insulin, pmoL/L, mean (SD)	40.86 (42.56)	40.04 (48.68)	34.88 (38.23)	38.58 (43.82)	31.65 (30.16)	32.44 (25.80)	<0.001
Proinsulin, pmoL/L, mean (SD)	9.47 (12.54)	8.88 (9.38)	8.64 (8.87)	9.20 (12.38)	7.70 (8.61)	7.30 (5.26)	<0.001
Triglycerides, mmol/L, mean (SD)	1.09 (.57)	1.04 (.49)	0.97 (0.46)	1.04 (0.59)	0 .97 (0.49)	0 .98 (0.49)	<0.001
HDL, mmol/L, mean (SD) ||	1.40 (.37)	1.41 (.36)	1.50 (0.38)	1.47 (0 .37)	1.54 (0.39)	1.52 (0.38)	<0.001
LDL, mmol/L, mean (SD) #	3.02 (.84)	3.00 (.86)	2.93 (0.78)	3.01 (0 .76)	2.92 (0.78)	2.99 (0 .81)	0.250
C-reactive protein, mg/L, mean (SD)	3.00 (4.65)	2.49 (3.73)	2.28 (3.53)	2.02 (3.59)	1.81 (3.00)	1.89 (4.03)	<0.001

*p-value coresponding to an unadjusted F test for any difference between breastfeeding categories compared to never breastfeeding
† SBP- systolic blood pressure
‡ DBP- diastolic blood pressure
§ cMIT - carotid intima-media thickness
|| HDL- high density lipoprotein
LDL- low density lipoprotein

Table S7. Test for linear trend for all participants for cardiometabolic outcomes with no evidence of a difference in the association with breastfeeding according to hypertensive disorders during pregnancy status

Outcome	Test for linear trend p-value
Triglycerides	0.013
HDL-cholesterol*	0.010
Body-mass index	0.011
Waist circumference	0.009
cIMT†	0.294
Arterial distensability	0.756
Insulin	0.022
Proinsulin	0.009
C-reactive protein	0.002
Systolic blood pressure	0.290

*HDL- high density lipoprotein
†cMIT - carotid intima-media thickness
The p-values were estimated by entering breastfeeding duration categories as a single continuous variable

Table S8. Test for linear trend for outcomes with evidence of a difference in the association with breastfeeding according to hypertensive disorders during pregnancy status

Outcome	Test for linear trend p-value among women without hypertensive disorders during pregnancy	Test for linear trend p-value among women with hypertensive disorders during pregnancy
LDL-cholesterol*	0.118	0.027
Diastolic blood pressure	0.122	0.174
Mean arterial pressure	0.234	0.108
Glucose	0.871	0.123

*LDL- low density lipoprotein

The p-values were estimated by entering breastfeeding duration categories as a single continuous variable

Table S9. Unadjusted associations of duration of any breastfeeding with measures of cardiometabolic health

	< 1 month	1-<3 months	3-<6 months	6- <9 months	9+ months
	Mean difference (95% CI)	Mean difference (95% CI)	Mean difference (95% CI)	Mean difference (95% CI)	Mean difference (95% CI)
Body Mass Index (kg/m2)	-0.98 (-1.70, -0.26)*	-1.09 (-1.75, -0.43)†	-1.42 (-2.01, -0.82)†	-2.22 (-2.82, -1.63)†	-2.28 (-2.82, -1.74)†
Waist Circumference (cm)	-1.79 (-3.47, -0.11)*	-2.69 (-4.23, -1.14) †	-3.09 (-4.48, -1.70) †	-4.72 (-6.12, -3.33) †	-4.73 (-6.00, -3.46) †
Systolic BP (mmHg)	-1.08 (-2.81, 0.65)	-1.88 (-3.47, -0.29)*	-0.68 (-2.11, 0.75)	-1.26 (-2.70, 0.17)	-1.59 (-2.90, -0.28)*
Arterial distensibility (mm)	-0.00 (-0.02, 0.01)	0.00 (-0.01, 0.02)	-0.01 (-0.02, 0.01)	-0.01 (-0.02, 0.01)	-0.01 (-0.03, -0.00) *
cIMT(mm) ‡	-0.01 (-0.02, 0.00)	-0.00 (-0.01, 0.01)	0.01 (-0.00, 0.01)	0.01 (0.00, 0.02)*	0.01 (0.00, 0.01)*
CRP (% change) §	-23.90 (-40.52, -7.27)*	-26.31(-41.64,-10.99)†	-33.95(-47.60,-20.29)†	-45.76(-59.75,-31.76)†	-42.80(-55.27,-30.34)†
HDL (mmol/L) ||	0.01 (-0.04, 0.07)	0.09 (0.04, 0.15)†	0.08 (0.03, 0.12)*	0.14 (0.10, 0.19)†	0.13 (0.09, 0.17)†
Triglycerides (mmol/L)	-0.07 (-0.14, 0.01)	-0.13 (-0.20, -0.06)†	-0.06 (-0.12, 0.00)	-0.12 (-0.18, -0.06)†	-0.12 (-0.17, -0.06)†
Insulin (% change)	-5.44 (-14.29, 3.40)	-14.42 (-22.81, -6.04)†	-9.54 (-17.02, -2.06)*	-21.28(-28.69,-13.86)†	-18.98(-25.67,-12.30)†
Proinsulin (% change)	-5.63 (-14.11, 2.85)	-10.44 (-18.33, -2.56)*	-6.55 (-13.38, 0.29)	-17.50(-24.35,-10.64)†	-16.04 (-22.39, -9.70)†

*p<.05
†p<.001 (Bonferroni correction)
‡ cMIT - carotid intima-media thickness
§ CRP- C- reactive protein
|| HDL- high density lipoprotein
Reference group=participants who never breastfed

Table S10. Unadjusted associations of duration of any breastfeeding with diastolic blood pressure, mean arterial pressure, and LDL-cholesterol stratified by hypertension during pregnancy

	< 1 month	1-<3 months	3-<6 months	6- <9 months	9+ months
	Mean difference (95% CI)	Mean difference (95% CI)	Mean difference (95% CI)	Mean difference (95% CI)	Mean difference (95% CI)
No Hypertensive disorders of pregnancy					
Diastolic BP (mmHg)	-0.42 (-1.97, 1.13)	-1.63 (-3.07, -0.19)*	-0.38 (-1.66, 0.90)	-0.90 (-2.18, 0.39)	-1.63 (-2.81, -0.45)*
Mean arterial pressure	-0.39 (-1.88, 1.11)	-1.55 (-2.95, -0.15)*	-0.12 (-1.36, 1.12)	-0.72 (-1.97, 0.53)	-1.34 (-2.49, -0.20)*
LDL cholesterol (mmol/L) ‡	0.02 (-0.12, 0.15)	-0.01 (-0.13, 0.12)	0.02 (-0.09, 0.13)	-0.05 (-0.16, 0.06)	-0.00 (-0.10, 0.10)
Glucose (% change)	-0.89 (-2.77, 0.98)	-1.31 (-3.05, 0.43)	-0.01 (-1.54, 1.53)	-1.65 (-3.18, -0.12)*	-0.51 (-1.90, 0.88)
Hypertensive disorders of pregnancy					
Diastolic BP (mmHg)	-4.26 (-7.91, -0.60)*	-3.53 (-6.68, -0.38)*	-3.18 (-6.30, -0.07)*	-5.04(-7.99,-2.10)†	-1.77 (-4.56, 1.02)
Mean arterial pressure	-3.81 (-7.33, -0.30)*	-3.69 (-6.72, -0.66)*	-3.28 (-6.28, -0.29)*	-4.73(-7.57,-1.89)†	-1.85 (-4.54, 0.83)
LDL cholesterol (mmol/L) ‡	-0.23 (-0.52, 0.05)	-0.39 (-0.65, -0.14)*	-0.12 (-0.36, 0.12)	-0.32 (-0.55, -0.09)*	-0.13 (-0.35, 0.09)
Glucose (% change)	-2.07 (-8.39, 4.25)	-6.11(-11.61,-0.60)*	-4.50 (-10.02, 1.03)	-6.50(-11.69,-1.31)*	-5.76(-10.71,-0.82)*

*p<.05

†p<.001 (Bonferroni correction)

‡LDL- low density lipoprotein

Reference group=participants who never breastfed

Table S11. Adjusted associations of duration of exclusive breastfeeding with measures of cardiometabolic health

	< 1 month	1-<3 months	3-<6 months
	Mean difference (95% CI)	Mean difference (95% CI)	Mean difference (95% CI)
Body Mass Index (kg/m2)	-0.28 (-0.65, 0.09)	-0.37 (-0.75, 0.01)	-0.46 (-0.82, -0.10)*
Waist Circumference (cm)	-0.80 (-1.79, 0.19)	-0.79 (-1.80, 0.22)	-1.27 (-2.23, -0.31)*
Systolic BP (mmHg)	-0.60 (-1.91, .71)	-.31 (-1.64, 1.02)	-0.94 (-2.20, 0.32)
Arterial distensibility (mm)	0.00 (-0.01, 0.02)	-0.00 (-0.01, 0.01)	0.00 (-0.01, 0.02)
cIMT(mm) ‡	-0.00 (-0.01, 0.00)	-0.00 (-0.01, 0.01)	0.00 (-0.01, 0.01)
CRP (% change) §	-16.01 (-28.23, -3.79)*	-26.59(-39.35, -13.82)†	-20.03(-31.86, -8.20)†
HDL (mmol/L) ||	0.03 (-0.01, 0.07)	0.05 (0.00, 0.08)*	0.04 (0.00, 0.08)*
Triglycerides (mmol/L)	-0.05 (-0.11, 0.01)	-0.07 (-0.13, -0.02)*	-0.06 (-0.12, -0.01)*
Insulin (% change)	-2.80 (-9.47, 3.86)	-8.49 (-15.17, -1.82)*	-7.04 (-13.41, -.67)*
Proinsulin (% change)	-3.77 (-9.98, 2.44)	-5.90 (-12.06, .25)	-4.27 (-10.16, 1.61)

*p<.05
†p<.001 (Bonferroni correction)
‡ cMIT - carotid intima-media thickness
§ CRP- C- reactive protein
|| HDL- high density lipoprotein
The reference group includes those who never breastfed. All outcomes were adjusted for age at delivery, race, pre-pregnancy BMI, parity, smoking, marital status, and gestational diabetes. Blood pressure outcomes were further adjusted for use of antihypertensive medications. Glucose, insulin, and proinsulin outcomes were further adjusted for diabetes medications. LDL-cholesterol and HDL-cholesterol outcomes were further adjusted for cholesterol lowering medications.

Table S12. P-values for adjusted associations of duration of exclusive breastfeeding with measures of cardiometabolic health

	< 1 month	1-<3 months	3-<6 months
Body Mass Index (kg/m2)	.14	.06	.01
Waist Circumference (cm)	.12	.12	.009
Systolic BP (mmHg)	.37	.65	.14
Arterial distensibility (mm)	.54	.83	.58
cIMT(mm) *	.51	.60	.72
CRP (% change) †	.01	.000	.001
HDL (mmol/L) ‡	.14	.04	.04
Triglycerides (mmol/L)	.10	.01	.03
Insulin (% change)	.41	.01	.03
Proinsulin (% change)	.23	.06	.16

*cMIT - carotid intima-media thickness
†CRP- C- reactive protein
‡HDL- high density lipoprotein

Table 13. P-values from the Interaction Test of Exclusive Breastfeeding and Hypertensive Disorders of Pregnancy on Cardiometabolic Health Measurements

Outcome	< 1 month	1-<3 months	3- <6 months
Body-mass index	0.629	0.261	0.917
Waist circumference	0.712	0.789	0.830
Systolic blood pressure	0.539	0.059	0.380
Diastolic blood pressure	0.301	0.050	0.587
Mean arterial pressure	0.335	0.034	0.465
cIMT*	0.417	0.727	0.191
Arterial distensibility	0.531	0.741	0.472
Glucose	0.539	0.005	0.043
Insulin	0.519	0.665	0.653
Proinsulin	0.970	0.262	0.763
Triglycerides	0.274	0.742	0.675
HDL-cholesterol†	0.217	0.588	0.998
LDL-cholesterol‡	0.048	0.028	0.042
C-reactive protein	0.712	0.788	0.924

*cMIT - carotid intima-media thickness

†HDL- high density lipoprotein

‡LDL- low density lipoprotein

p <.05 indicates a significant interaction test between exclusive breastfeeding by duration category and hypertensive disorder of pregnancy

Table S14. Adjusted associations of duration of exclusive breastfeeding with diastolic blood pressure, mean arterial pressure, LDL-cholesterol, and glucose stratified by the presence of hypertension during pregnancy

	< 1 month	1-<3 months	3-<6 months
	Mean difference (95% CI)	Mean difference (95% CI)	Mean difference (95% CI)
No Hypertensive disorders of pregnancy			
Diastolic BP (mmHg)	-0.56 (-1.77, 0.66)	-0.71 (-1.94, 0.52)	-0.65 (-1.82, 0.52)
Mean arterial pressure	-0.43 (-1.60, 0.73)	-0.32 (-1.50, 0.86)	-0.53 (-1.65, 0.59)
LDL cholesterol (mmol/L) ‡	-0.01 (-0.11, 0.10)	0.02 (-0.08, 0.12)	-0.05 (-0.15, 0.05)
Glucose (% change)	-0.46 (-1.79, 0.88)	0.36 (-0.93, 1.65)	-0.24 (-1.48, 1.00)
Hypertensive disorders of pregnancy			
Diastolic BP (mmHg)	-2.58 (-5.30, 0.14)	-4.22 (-7.04, -1.40)*	-2.63 (-5.34, 0.09)
Mean arterial pressure	-2.44 (-5.01, 0.14)	-4.07 (-6.74, -1.40)*	-2.94 (-5.51, -0.37)*
LDL cholesterol (mmol/L) ‡	-0.29 (-0.50, -0.08)*	-0.32 (-0.54, -0.10)*	-0.35 (-0.55, -0.14)†
Glucose (% change)	-1.78 (-5.49, 1.93)	-4.48 (-8.35, -0.61)*	-3.44 (-7.17, 0.29)

*p<.05
†p<.001 (Bonferroni correction)
‡ LDL- low density lipoprotein
The reference group includes those who never breastfed. All outcomes were adjusted for age at delivery, race, pre-pregnancy BMI, parity, smoking, marital status, and gestational diabetes. Blood pressure outcomes were further adjusted for use of antihypertensive medications. Glucose, insulin, and proinsulin outcomes were further adjusted for diabetes medications. LDL-cholesterol and HDL-cholesterol outcomes were further adjusted for cholesterol lowering medications.

Table S15. p values for adjusted associations of duration of exclusive breastfeeding with diastolic blood pressure, mean arterial pressure, LDL-cholesterol, and glucose stratified by the presence of hypertension during pregnancy

	< 1 month	1-<3 months	3-<6 months
No Hypertensive disorders of pregnancy			
Diastolic BP (mmHg)	.37	.26	.27
Mean arterial pressure	.47	.60	.35
LDL cholesterol (mmol/L)*	.88	.70	.35
Glucose (% change)	.50	.58	.71
Hypertensive disorders of pregnancy			
Diastolic BP (mmHg)	.06	.004	.06
Mean arterial pressure	.06	.003	.03
LDL cholesterol (mmol/L)*	.008	.005	.001
Glucose (% change)	.35	.02	.07

*LDL- low density lipoprotein

Table S16. Unadjusted associations of duration of exclusive breastfeeding with measures of cardiometabolic health

	< 1 month	1-<3 months	3-<6 months
	Mean difference (95% CI)	Mean difference (95% CI)	Mean difference (95% CI)
Body Mass Index (kg/m2)	-1.05 (-1.61, -0.49)†	-1.61 (-2.18, -1.05)†	-2.35 (-2.87, -1.83)†
Waist Circumference (cm)	-2.36 (-3.67, -1.05)†	-3.29 (-4.61, -1.96)†	-5.01 (-6.22, -3.79)†
Systolic BP (mmHg)	-0.94 (-2.29, 0.42)	-1.02 (-2.38, 0.35)	-1.73 (-2.98, -0.48)*
Arterial distensibility (mm)	-0.00 (-0.02, 0.01)	-0.01 (-0.02, 0.01)	-0.01 (-0.02, 0.00)
cIMT(mm) ‡	0.00 (-0.01, 0.01)	0.00 (-0.01, 0.01)	0.01 (0.00, 0.01)*
CRP (% change) §	-24.80 (-37.66, -11.92)†	-41.01 (-54.32, -27.67)†	-42.99 (-55.04, -30.95)†
HDL (mmol/L) ||	0.07 (0.03, 0.12)†	0.10 (0.05, 0.14)†	0.13 (0.09, 0.17)†
Triglycerides (mmol/L)	-0.07 (-0.13, -0.02)*	-0.11 (-0.17, -0.05)†	-0.11 (-0.17, -0.06)†
Insulin (% change)	-8.01 (-15.01, -1.02)*	-16.47 (-23.47, -9.47)†	-19.51 (-25.98, -13.03)†
Proinsulin (% change)	-8.40 (-14.92, -1.87)	-13.17 (-19.78, -6.56)	-14.67 (-20.72, -8.62)

*p<.05
†p<.001 (Bonferroni correction)
‡ cMIT - carotid intima-media thickness
§ CRP- C- reactive protein
|| HDL- high density lipoprotein
The reference group includes those who never breastfed.

Table S17. Unadjusted associations of duration of exclusive breastfeeding with diastolic blood pressure, mean arterial pressure, LDL-cholesterol, and glucose stratified by hypertension during pregnancy

	< 1 month	1-<3 months	3-<6 months
	Mean difference (95% CI)	Mean difference (95% CI)	Mean difference (95% CI)
No Hypertensive disorders of pregnancy			
Diastolic BP (mmHg)	-0.75 (-1.97, 0.47)	-1.15 (-2.38, 0.09)	-1.25 (-2.38, -0.12)*
Mean arterial pressure	-0.63 (-1.81, 0.56)	-0.76 (-1.95, 0.43)	-1.11 (-2.20, -0.01)*
LDL cholesterol (mmol/L) ‡	0.00 (-0.10, 0.11)	0.02 (-0.09, 0.12)	-0.03 (-0.12, 0.07)
Glucose (% change)	-0.93 (-2.40, 0.55)	-0.11 (-1.56, 1.35)	-1.09 (-2.43, 0.25)
Hypertensive disorders of pregnancy			
Diastolic BP (mmHg)	-2.93 (-5.68, -0.18)*	-4.82 (-7.64, -2.00)†	-2.70 (-5.33, -0.07)*
Mean arterial pressure	-2.69 (-5.34, -0.05)*	-4.67 (-7.38, -1.96)†	-2.87 (-5.40, -0.35)*
LDL cholesterol (mmol/L) ‡	-0.23 (-0.45, -0.01)*	-0.23 (-0.45, -0.00)*	-0.24 (-0.44, -0.03)*
Glucose (% change)	-2.36 (-7.18, 2.47)	-7.92 (-12.90, -2.95)*	-5.87 (-10.51, -1.23)*

*p<.05

†p<.001 (Bonferroni correction)

‡LDL- low density lipoprotein

The reference group includes those who never breastfed.

Table S18. Duration of Breastfeeding (months) according to presence of hypertensive disorders of pregnancy (HDPs) and preterm birth

Breastfeeding outcome	Category	No HDP or Preterm Birth (N=2,959)	Preterm Birth Only (n=110)	HDP Only (n=495)	HDP and preterm birth (n=34)
Any Breastfeeding Duration	Never breastfed, % (n)	13.5 (398)	16.4 (18)	17.8 (88)	14.7 (5)
	<1, % (n)	8.5 (253)	15.5(17)	8.7 (43)	11.8 (4)
	1-<3, % (n)	11.6 (342)	7.3 (8)	14.7 (73)	14.7 (5)
	3-<6, % (n)	18.5 (548)	19.1 (21)	16.4 (81)	8.8 (3)
	6-<9, % (n)	18.0 (532)	17.3 (19)	18.0 (89)	32.4 (11)
	9+, % (n)	30.0 (886)	24.6 (27)	24.4 (121)	17.7 (6)
Exclusive breastfeeding Duration	Never breastfed, % (n)	13.5 (398)	16.4 (18)	17.8 (88)	14.7 (5)
	0-<1, % (n)	23.6 (698)	32.7 (36)	25.5 (126)	32.4 (11)
	1-<3, % (n)	23.3 (687)	18.2 (20)	23.0 (114)	29.4 (10)
	3-6, % (n)	39.7 (1,176)	32.7 (36)	33.7 (167)	23.5 (8)

Duration of exclusive breastfeeding is likely to differ from duration of any breastfeeding (i.e., a participant exclusively breastfed for 2 months but continued to breastfeed until 6 months). Therefore the percentage and number of individuals in each breastfeeding category cannot be compared between any and exclusive breastfeeding

Table S19. Inverse probability weighting results for associations of any breastfeeding with cardiometabolic outcomes

	< 1 month	1-<3 months	3-<6 months	6- <9 months	9+ months
	Mean difference (95% CI)	Mean difference (95% CI)	Mean difference (95% CI)	Mean difference (95% CI)	Mean difference (95% CI)
Body Mass Index (kg/m2)	-0.44 (-1.03, 0.15)	-0.18 (-0.67, 0.30)	-0.00 (-0.47, 0.46)	-0.40 (-0.85, 0.05)	-0.41 (-0.83, -0.00)
Waist Circumference (cm)	-0.38 (-1.91, 1.16)	-0.67 (-1.91, 0.57)	-0.18 (-1.35, 0.98)	-1.07 (-2.21, 0.06)	-1.07 (-2.13, -0.00)
Systolic BP (mmHg)	-0.48 (-2.02, 1.06)	-1.74 (-3.23, -0.26)	-0.74 (-2.10, 0.62)	-1.45 (-2.79, -0.12)	-2.11 (-3.39, -0.84)
Arterial distensibility (mm)	0.00 (-0.01, 0.02)	0.01 (-0.01, 0.02)	0.00 (-0.01, 0.02)	0.01 (-0.01, 0.02)	0.00 (-0.01, 0.02)
cIMT(mm) *	-0.01 (-0.01, 0.00)	-0.00 (-0.01, 0.01)	0.00 (-0.01, 0.01)	0.00 (-0.00, 0.01)	-0.00 (-0.01, 0.01)
CRP (% change) †	-11.31 (-28.13, 5.51)	-6.41 (-20.88, 8.07)	-11.86 (-24.72, 1.01)	-19.99 (-33.74, -6.24)	-13.95 (-26.20, -1.69)
HDL (mmol/L) ‡	-0.01 (-0.06, 0.04)	0.05 (0.00, 0.10)	0.02 (-0.02, 0.06)	0.08 (0.04, 0.13)	0.04 (-0.00, 0.08)
Triglycerides (mmol/L)	-0.08 (-0.18, 0.01)	-0.10 (-0.19, -0.01)	-0.07 (-0.16, 0.01)	-0.13 (-0.21, -0.06)	-0.13 (-0.20, -0.05)
Insulin (% change)	-0.05 (-8.94, 8.85)	-4.23 (-12.06, 3.60)	3.00 (-4.47, 10.47)	-8.04 (-15.61, -0.47)	-4.41 (-11.02, 2.21)
Proinsulin (% change)	-1.57 (-10.46, 7.31)	-0.35 (-8.79, 8.09)	1.67 (-6.39, 9.74)	-4.35 (-12.66, 3.95)	-4.80 (-11.55, 1.95)

*cMIT - carotid intima-media thickness

†CRP- C- reactive protein

‡HDL- high density lipoprotein

The reference group includes those who never breastfed

All outcomes were adjusted for age at delivery, race, pre-pregnancy BMI, smoking, marital status, and gestational diabetes. Blood pressure outcomes were further adjusted for use of antihypertensive medications. Glucose, insulin, and proinsulin outcomes were further adjusted for diabetes medications. LDL-cholesterol and HDL-cholesterol outcomes were further adjusted for cholesterol lowering medications.

Inverse probability weighting was further used to examine the potential role of selection bias due to the participation rate at the 18 years follow-up visit. We estimated the probability of attending the follow-up visit 18 years after recruitment according to age, parity, race, marital status, social class, educational level, self-reported pre-pregnancy BMI, smoking status, HDP, and preterm birth in the index pregnancy and used these to weight analyses.

Table S20. Inverse probability weighting results for associations of any breastfeeding with cardiometabolic outcomes stratified by hypertension during pregnancy

	< 1 month	1-<3 months	3-<6 months	6- <9 months	9+ months
	Mean difference (95% CI)	Mean difference (95% CI)	Mean difference (95% CI)	Mean difference (95% CI)	Mean difference (95% CI)
No Hypertensive disorders of pregnancy					
Diastolic BP (mmHg)	-0.14 (-1.60, 1.33)	-1.26 (-2.65, 0.12)	-0.51 (-1.76, 0.75)	-1.24 (-2.53, 0.05)	-1.70 (-2.90, -0.50)
Mean arterial pressure	-0.21 (-1.61, 1.19)	-1.16 (-2.48, 0.17)	-0.30 (-1.50, 0.90)	-1.04 (-2.28, 0.19)	-1.61 (-2.75, -0.47)
LDL cholesterol (mmol/L)*	-0.01 (-0.13, 0.12)	0.02 (-0.10, 0.13)	0.01 (-0.09, 0.11)	-0.08 (-0.18, 0.03)	-0.02 (-0.12, 0.08)
Glucose (% change)	0.08 (-1.60, 1.76)	-1.02 (-2.60, 0.57)	0.72 (-0.72, 2.15)	-0.48 (-1.96, 1.00)	0.32 (-1.05, 1.68)
Hypertensive disorders of pregnancy					
Diastolic BP (mmHg)	-3.14 (-6.56, 0.28)	-4.00 (-7.00, -1.00)	-3.02 (-6.05, 0.01)	-4.36 (-7.30, -1.43)	-1.78 (-4.66, 1.10)
Mean arterial pressure	-3.01 (-6.22, 0.19)	-4.25 (-7.06, -1.43)	-3.26 (-6.10, -0.41)	-4.32 (-7.07, -1.57)	-2.17 (-4.87, 0.54)
LDL cholesterol (mmol/L)*	-0.25 (-0.52, 0.02)	-0.26 (-0.50, -0.02)	-0.11 (-0.36, 0.13)	-0.31 (-0.55, -0.08)	-0.16 (-0.39, 0.07)
Glucose (% change)	-1.48 (-6.25, 3.30)	-2.75 (-7.05, 1.55)	-1.33 (-5.60, 2.95)	-3.53 (-7.67, 0.62)	-2.65 (-6.77, 1.48)

*LDL- low density lipoprotein

Reference group=participants who never breastfed

All outcomes were adjusted for age at delivery, race, pre-pregnancy BMI, smoking, marital status, and gestational diabetes. Blood pressure outcomes were further adjusted for use of antihypertensive medications. Glucose, insulin, and proinsulin outcomes were further adjusted for diabetes medications. LDL-cholesterol and HDL-cholesterol outcomes were further adjusted for cholesterol lowering medications.

Inverse probability weighting was further used to examine the potential role of selection bias due to the participation rate at the 18 years follow-up visit. We estimated the probability of attending the follow-up visit 18 years after recruitment according to age, parity, race, marital status, social class, educational level, self-reported pre-pregnancy BMI, smoking status, HDP, and preterm birth in the index pregnancy and used these to weight analyses.

Table S21. Inverse probability weighting results for associations of exclusive breastfeeding with cardiometabolic outcomes

	< 1 month	1-<3 months	3-<6 months
	Mean difference (95% CI)	Mean difference (95% CI)	Mean difference (95% CI)
Body Mass Index (kg/m2)	-0.24 (-0.62, 0.15)	-0.09 (-0.48, 0.30)	-0.40 (-0.77, -0.02)
Waist Circumference (cm)	-0.46 (-1.50, 0.57)	-0.27 (-1.32, 0.79)	-0.94 (-1.94, 0.07)
Systolic BP (mmHg)	0.13 (-1.23, 1.50)	0.23 (-1.17, 1.62)	-0.53 (-1.86, 0.80)
Arterial distensibility (mm)	0.00 (-0.01, 0.01)	-0.00 (-0.02, 0.01)	0.00 (-0.01, 0.02)
cIMT(mm) *	0.00 (-0.01, 0.01)	-0.00 (-0.01, 0.01)	0.00 (-0.00, 0.01)
CRP (% change) †	-10.34 (-23.63, 2.95)	-19.66 (-33.15, -6.17)	-13.51 (-26.42, -0.60)
HDL (mmol/L) ‡	0.01 (-0.03, 0.06)	0.04 (-0.00, 0.09)	0.04 (-0.00, 0.09)
Triglycerides (mmol/L)	-0.01 (-0.08, 0.05)	-0.06 (-0.12, 0.00)	-0.05 (-0.11, 0.01)
Insulin (% change)	2.72 (-4.39, 9.84)	-1.68 (-8.89, 5.54)	-1.98 (-8.89, 4.93)
Proinsulin (% change)	0.64 (-6.35, 7.64)	0.86 (-6.25, 7.98)	0.33 (-6.47, 7.13)

*cMIT - carotid intima-media thickness

†CRP- C- reactive protein

‡HDL- high density lipoprotein

Reference group=participants who never breastfed

All outcomes were adjusted for age at delivery, race, pre-pregnancy BMI, smoking, marital status, and gestational diabetes. Blood pressure outcomes were further adjusted for use of antihypertensive medications. Glucose, insulin, and proinsulin outcomes were further adjusted for diabetes medications. LDL-cholesterol and HDL-cholesterol outcomes were further adjusted for cholesterol lowering medications.

Inverse probability weighting was further used to examine the potential role of selection bias due to the participation rate at the 18 years follow-up visit. We estimated the probability of attending the follow-up visit 18 years after recruitment according to age, parity, race, marital status, social class, educational level, self-reported pre-pregnancy BMI, smoking status, HDP, and preterm birth in the index pregnancy and used these to weight analyses.

Table S22. Inverse probability weighting results for associations of exclusive breastfeeding with cardiometabolic outcomes stratified by hypertension during pregnancy

	< 1 month	1-<3 months	3-<6 months
	Mean difference (95% CI)	Mean difference (95% CI)	Mean difference (95% CI)
No Hypertensive disorders of pregnancy			
Diastolic BP (mmHg)	-0.54 (-172, 0.65)	-0.98 (-2.19, 0.23)	-0.81 (-1.96, 0.34)
Mean arterial pressure	-0.31 (-1.45, 0.82)	-0.58 (-1.73, 0.58)	-0.72 (-1.82, 0.39)
LDL cholesterol (mmol/L)*	-0.02 (-0.12, 0.08)	0.00 (-0.10, 0.11)	-0.03 (-0.13, 0.06)
Glucose (% change)	-0.33 (-1.70, 1.04)	0.19 (-1.20, 1.58)	-0.03 (-1.37, 1.30)
Hypertensive disorders of pregnancy			
Diastolic BP (mmHg)	-2.90 (-6.09, 0.28)	-4.20 (-7.36, -1.04)	-1.39 (-4.56, 1.77)
Mean arterial pressure	-2.74 (-5.90, 0.43)	-3.77 (-6.92, -0.63)	-1.13 (-4.27, 2.01)
LDL cholesterol (mmol/L)	-0.51 (-0.82, -0.20)	-0.34 (0.66, -0.02)	-0.46 (-0.78, -0.15)
Glucose (% change)	-1.63 (-7.39, 4.12)	-4.63 (-10.39, 1.14)	-2.58 (-8.29, 3.14)

*LDL- low density lipoprotein
The reference group includes those who never breastfed
All outcomes were adjusted for age at delivery, race, pre-pregnancy BMI, smoking, marital status, and gestational diabetes. Blood pressure outcomes were further adjusted for use of antihypertensive medications. Glucose, insulin, and proinsulin outcomes were further adjusted for diabetes medications. LDL-cholesterol and HDL-cholesterol outcomes were further adjusted for cholesterol lowering medications.

Inverse probability weighting was further used to examine the potential role of selection bias due to the participation rate at the 18 years follow-up visit. We estimated the probability of attending the follow-up visit 18 years after recruitment according to age, parity, race, marital status, social class, educational level, self-reported pre-pregnancy BMI, smoking status, HDP, and preterm birth in the index pregnancy and used these to weight analyses.

Table S23. Sensitivity analysis for associations of any breastfeeding with cardiometabolic outcomes restricted to primiparous women

	< 1 month	1-<3 months	3-<6 months	6- <9 months	9+ months
	Mean difference (95% CI)	Mean difference (95% CI)	Mean difference (95% CI)	Mean difference (95% CI)	Mean difference (95% CI)
Body Mass Index (kg/m2)	-0.36 (-1.11, 0.39)	-0.67 (-1.39, 0.04)	-0.30 (-0.95, 0.35)	-0.67 (-1.35, -0.00)	-0.65 (-1.31, 0.01)
Waist Circumference (cm)	-0.23 (-2.19, 1.74)	-1.82 (-3.69, 0.06)	-1.09 (-2.80, 0.61)	-1.99 (-3.75, -0.23)	-2.50 (-4.23, -0.77)
Systolic BP (mmHg)	-0.59 (-3.17, 1.99)	-1.18 (-3.63, 1.27)	-0.06 (-2.29, 2.16)	0.23 (-2.08, 2.54)	-0.89 (-3.15, 1.38)
Arterial distensibility (mm)	-0.02 (-0.05, 0.00)	-0.00 (-0.03, 0.02)	-0.01 (-0.03, 0.01)	-0.00 (-0.03, 0.02)	-0.01 (-0.03, 0.01)
cIMT(mm) *	-0.02 (-0.03, -0.00)	-0.01 (-0.02, 0.01)	-0.00 (-0.01, 0.01)	0.00 (-0.01, 0.01)	-0.01 (-0.02, 0.00)
CRP (% change) †	-16.01 (-41.32, 9.29)	-28.51 (-52.70, -4.31)	-22.81 (-44.68, -0.95)	-26.50 (-49.45, -3.55)	-20.77 (-42.80, 1.25)
HDL (mmol/L) ‡	-0.05 (-0.14, 0.04)	0.02 (-0.06, 0.11)	0.00 (-0.08, 0.08)	0.09 (0.01, 0.17)	0.03 (-0.05, 0.11)
Triglycerides (mmol/L)	0.08 (-0.03, 0.19)	-0.04 (-0.14, 0.05)	0.02 (-0.08, 0.12)	-0.04 (-0.14, 0.05)	-0.06 (-0.16, 0.04)
Insulin (% change)	0.88 (-12.62, 14.38)	-10.39 (-23.52, 2.75)	-4.84 (-16.76, 7.09)	-8.82 (-21.23, 3.60)	-10.31 (-22.26, 1.65)
Proinsulin (% change)	-1.75 (-14.26, 10.76)	-10.96 (-22.74, 0.83)	-5.06 (-15.62, 5.50)	-6.79 (-17.62, 4.04)	-12.70 (-23.16, -2.25)

*cMIT - carotid intima-media thickness

†CRP- C- reactive protein

‡HDL- high density lipoprotein

Reference group=participants who never breastfed

All outcomes were adjusted for age at delivery, race, pre-pregnancy BMI, smoking, marital status, and gestational diabetes. Blood pressure outcomes were further adjusted for use of antihypertensive medications. Glucose, insulin, and proinsulin outcomes were further adjusted for diabetes medications. LDL-cholesterol and HDL-cholesterol outcomes were further adjusted for cholesterol lowering medications.

Table S24. Sensitivity analysis for associations of any breastfeeding with cardiometabolic outcomes by hypertension during pregnancy restricted to primiparous women

	< 1 month	1-<3 months	3-<6 months	6- <9 months	9+ months
	Mean difference (95% CI)	Mean difference (95% CI)	Mean difference (95% CI)	Mean difference (95% CI)	Mean difference (95% CI)
No Hypertensive disorders of pregnancy					
Diastolic BP (mmHg)	-0.87 (-3.34, 1.60)	-1.50 (-3.95, 0.95)	0.78 (-1.40, 2.95)	-0.04 (-2.29, 2.22)	-2.55 (-4.76, -0.35)
Mean arterial pressure	-0.57 (-2.93, 1.79)	-1.28 (-3.62, 1.05)	0.97 (-1.10, 3.04)	0.36 (-1.79, 2.51)	-1.74 (-3.84, 0.36)
LDL cholesterol (mmol/L)*	0.13 (-0.09, 0.35)	0.10 (-0.11, 0.31)	0.08 (-0.11, 0.28)	-0.04 (-0.23, 0.16)	-0.06 (-0.25, 0.13)
Glucose (% change)	0.51 (-1.95, 2.97)	-0.10 (-2.57, 2.37)	1.05 (-1.16, 3.27)	-0.03 (-2.29, 2.23)	-0.68 (-2.89, 1.53)
Hypertensive disorders of pregnancy					
Diastolic BP (mmHg)	-2.24 (-6.45, 1.97)	-2.94 (-6.35, 0.46)	-3.99 (-7.53, -0.45)	-3.71 (-7.19, -0.22)	-0.82 (-4.31, 2.68)
Mean arterial pressure	-1.36 (-5.55, 2.83)	-2.82 (-6.21, 0.56)	-3.88 (-7.39, -0.36)	-3.09 (-6.55, 0.38)_	-0.87 (-4.35, 2.60)
LDL cholesterol (mmol/L)*	-0.49 (-0.90, -0.08)	-0.47 (-0.82, -0.13)	-0.31 (-0.65, 0.03)	-0.54 (-0.88, -0.20)	-0.35 (-0.71, 0.01)
Glucose (% change)	-0.44 (-7.82, 6.94)	-6.38 (-12.47, -0.29)	-0.77 (-7.28, 5.73)	-3.26 (-9.33, 2.82)	-1.33 (-7.56, 4.90)

*LDL- low density lipoprotein

Reference group=participants who never breastfed

All outcomes were adjusted for age at delivery, race, pre-pregnancy BMI, smoking, marital status, and gestational diabetes. Blood pressure outcomes were further adjusted for use of antihypertensive medications. Glucose, insulin, and proinsulin outcomes were further adjusted for diabetes medications. LDL-cholesterol and HDL-cholesterol outcomes were further adjusted for cholesterol lowering medications.

Table S25. Sensitivity analysis for associations of exclusive breastfeeding with cardiometabolic outcomes restricted to primiparous women

	< 1 month	1-<3 months	3-<6 months
	Mean difference (95% CI)	Mean difference (95% CI)	Mean difference (95% CI)
Body Mass Index (kg/m2)	-0.05 (-0.75, 0.65)	-0.49 (-1.19, 0.22)	-0.60 (-1.29, 0.09)
Waist Circumference (cm)	-0.22 (2.08, 1.64)	-1.06 (-2.93, 0.81)	-1.79 (-3.62, 0.05)
Systolic BP (mmHg)	0.19 (-2.27, 2.64)	0.91 (-1.55, 3.36)	0.29 (-2.12, 2.70)
Arterial distensibility (mm)	-0.01 (-0.04, 0.01)	-0.01 (-0.03, 0.02)	-0.00 (-0.03, 0.02)
cIMT(mm) *	-0.01 (-0.02, 0.01)	-0.01 (-0.02, 0.01)	-0.00 (-0.01, 0.01)
CRP (% change) †	-3.12 (-27.08, 20.85)	-24.82 (-49.42, -0.22)	-19.34 (-43.28, 4.60)
HDL (mmol/L) ‡	-0.05 (-0.14, 0.03)	0.05 (-0.03, 0.13)	0.02 (-0.06, 0.11)
Triglycerides (mmol/L)	0.07 (-0.03, 0.17)	-0.08 (-0.18, 0.02)	0.01 (-0.09, 0.11)
Insulin (% change)	-2.21 (-15.08, 10.65)	-14.23 (-27.18, -1.29)	-7.59 (-20.08, 4.91)
Proinsulin (% change)	-2.71 (-14.68, 9.25)	-10.40 (-22.27, 1.48)	-5.05 (-16.16, 6.06)

*cMIT - carotid intima-media thickness
†CRP- C- reactive protein
‡HDL- high density lipoprotein
Reference group=participants who never breastfed
All outcomes were adjusted for age at delivery, race, pre-pregnancy BMI, smoking, marital status, and gestational diabetes. Blood pressure outcomes were further adjusted for use of antihypertensive medications. Glucose, insulin, and proinsulin outcomes were further adjusted for diabetes medications. LDL-cholesterol and HDL-cholesterol outcomes were further adjusted for cholesterol lowering medications.

Table S26. Sensitivity analysis for associations of exclusive breastfeeding with cardiometabolic outcomes by hypertension during pregnancy restricted to primiparous women

	< 1 month	1-<3 months	3-<6 months
	Mean difference (95% CI)	Mean difference (95% CI)	Mean difference (95% CI)
No Hypertensive disorders of pregnancy			
Diastolic BP (mmHg)	-1.04 (-3.15, 1.08)	-0.07 (-2.20, 2.06)	-1.01 (-3.10, 1.08)
Mean arterial pressure	-0.63 (-2.64, 1.38)	0.25 (-1.77, 2.28)	-0.58 (-2.57, 1.41)
LDL cholesterol (mmol/L)*	0.11 (-0.08, 0.29)	-0.01 (-0.19, 0.18)	0.01 (-0.17, 0.20)
Glucose (% change)	0.49 (-1.66, 2.63)	-0.36 (-1.76, 2.49)	-0.23 (-2.31,1.86)
Hypertensive disorders of pregnancy			
Diastolic BP (mmHg)	-2.90 (-6.09,0.28)	-4.20 (-7.36, -1.04)	-1.39 (-4.56, 1.77)
Mean arterial pressure	-2.74 (-5.90, 0.43)	-3.77 (-6.92, -0.63)	-1.13 (-4.27, 2.01)
LDL cholesterol (mmol/L)*	-0.51 (-0.81, -0.20)	-0.34 (-0.66, -0.02)	-0.46 (-0.78, -0.15)
Glucose (% change)	-1.63 (-7.39, 4.12)	-4.63 (-10.39, 1.14)	-2.58 (-8.29, 3.14)

*LDL- low density lipoprotein

Reference group=participants who never breastfed

All outcomes were adjusted for age at delivery, race, pre-pregnancy BMI, smoking, marital status, and gestational diabetes. Blood pressure outcomes were further adjusted for use of antihypertensive medications. Glucose, insulin, and proinsulin outcomes were further adjusted for diabetes medications. LDL-cholesterol and HDL-cholesterol outcomes were further adjusted for cholesterol lowering medications.

Table S27. Sensitivity analysis for associations of any breastfeeding with cardiometabolic outcomes restricted to multiparous women

	< 1 month	1-<3 months	3-<6 months	6- <9 months	9+ months
	Mean difference (95% CI)	Mean difference (95% CI)	Mean difference (95% CI)	Mean difference (95% CI)	Mean difference (95% CI)
Body Mass Index (kg/m2)	-0.32 (-0.94, 0.30)	-0.18 (-0.74, 0.38)	-0.09 (-0.60, -.42)	-0.53 (-1.04, -0.01)	-0.44 (-0.90, 0.03)
Waist Circumference (cm)	-0.64 (-2.31, 1.03)	-0.86 (-2.36, 0.63)	-0.37 (-1.74, 1.01)	-1.06 (-2.43, 0.31)	-0.75 (-1.99, 0.50)
Systolic BP (mmHg)	-0.40 (-2.59, 1.79)	-1.23 (-3.20, 0.74)	0.19 (-1.61, 1.98)	-0.78 (-2.59, 1.03)	-1.14 (-2.78, 0.50)
Arterial distensibility (mm)	0.01 (-0.01, 0.03)	0.01 (-0.01, 0.03)	0.00 (-0.01, 0.02)	0.01 (-0.01, 0.03)	0.01 (-0.01, 0.02)
cIMT(mm) *	-0.00 (-0.02, 0.01)	0.00 (-0.01, 0.01)	0.01 (-0.00, 0.01)	0.00 (-0.00, 0.01)	0.00 (-0.01, 0.01)
CRP (% change) †	-20.48 (-40.23, -0.72)	-9.77 (-27.63, 8.08)	-18.89 (-35.00, -2.78)	-25.31 (-41.68, -8.95)	-20.92 (-35.59, -6.24)
HDL (mmol/L) ‡	0.01 (-0.06, 0.08)	0.09 (0.03, 0.15)	0.04 (-0.02, 0.09)	0.06 (0.00, 0.11)	0.05 (-0.00, 0.10)
Triglycerides (mmol/L)	-0.10 (-0.20, 0.00)	-0.12 (-0.20, -0.03)	-0.04 (-0.12, 0.04)	-0.08 (-0.16, 0.01)	-0.09 (-0.16, -0.01)
Insulin (% change)	-3.64 (-14.49, 7.20)	-9.05 (-18.89, 0.79)	0.64 (-8.16, 9.45)	-11.94 (-20.70, -3.18)	-5.48 (-13.41, 2.46)
Proinsulin (% change)	-1.80 (-12.13, 8.54)	-2.45 (-11.65, 6.74)	3.79 (-4.51, 12.08)	-10.03 (-18.37, -1.69)	-4.92 (-12.46, 2.63)

*cMIT - carotid intima-media thickness
†CRP- C- reactive protein
‡HDL- high density lipoprotein
Reference group=participants who never breastfed
All outcomes were adjusted for age at delivery, race, pre-pregnancy BMI, smoking, marital status, and gestational diabetes. Blood pressure outcomes were further adjusted for use of antihypertensive medications. Glucose, insulin, and proinsulin outcomes were further adjusted for diabetes medications. LDL-cholesterol and HDL-cholesterol outcomes were further adjusted for cholesterol lowering medications.

Table S28. Sensitivity analysis for associations of any breastfeeding with cardiometabolic outcomes by hypertension during pregnancy restricted to multiparous women

	< 1 month	1-<3 months	3-<6 months	6- <9 months	9+ months
	Mean difference (95% CI)	Mean difference (95% CI)	Mean difference (95% CI)	Mean difference (95% CI)	Mean difference (95% CI)
No Hypertensive disorders of pregnancy					
Diastolic BP (mmHg)	0.24 (-1.74, 2.21)	-1.15 (-2.91, 0.61)	-0.59 (-2.20, 1.02)	-0.66 (-2.28, 0.95)	-0.73 (-2.20, 0.74)
Mean arterial pressure	0.16 (-1.74, 2.06)	-1.08 (-2.77, 0.62)	-0.22 (-1.76, 1.33)	-0.52 (-2.08, 1.04)	-0.75 (-2.16, 0.67)
LDL cholesterol (mmol/L)*	-0.05 (-0.21, 0.12)	-0.03 (-0.18, 0.12)	-0.04 (-0.17, 0.09)	-0.05 (-0.18, 0.08)	-0.05 (-0.17, 0.07)
Glucose (% change)	-0.73 (-2.88, 1.42)	-0.94 (-2.94, 1.06)	0.88 (-0.87, 2.62)	-0.93 (-2.68, 0.83)	0.14 (-1.44, 1.72)
Hypertensive disorders of pregnancy					
Diastolic BP (mmHg)	-4.47 (-10.28, 1.34)	-2.55 (-7.98, 2.88)	-2.49 (-7.62, 2.64)	-6.88 (-11.83, -1.94)	-2.71 (-7.37, 1.95)
Mean arterial pressure	-4.52 (-9.89, 0.85)	-2.82 (-7.84, 2.20)	-2.80 (-7.54, 1.95)	-6.75 (-11.32, -2.18)	-3.05 (-7.36, 1.26)
LDL cholesterol (mmol/L)*	-0.00 (-0.38, 0.38)	-0.30 (-0.66, 0.05)	-0.09 (-0.42, 0.25)	-0.25 (-0.56, 0.07)	-0.16 (-0.46, 0.14)
Glucose (% change)	-0.34 (-6.49, 5.82)	-0.78 (-6.76, 5.20)	-1.77 (-7.24, 3.70)	-4.01 (-9.37, 1.36)	-3.97 (-9.02, 1.08)

*LDL- low density lipoprotein

Reference group=participants who never breastfed

All outcomes were adjusted for age at delivery, race, pre-pregnancy BMI, smoking, marital status, and gestational diabetes. Blood pressure outcomes were further adjusted for use of antihypertensive medications. Glucose, insulin, and proinsulin outcomes were further adjusted for diabetes medications. LDL-cholesterol and HDL-cholesterol outcomes were further adjusted for cholesterol lowering medications.

Table S29. Sensitivity analysis for associations of exclusive breastfeeding with cardiometabolic outcomes restricted to multiparous women

	< 1 month	1-<3 months	3-<6 months
	Mean difference (95% CI)	Mean difference (95% CI)	Mean difference (95% CI)
Body Mass Index (kg/m2)	-0.33 (-0.82, 0.17)	-0.12 (-0.62, 0.39)	-0.40 (-0.86, 0.07)
Waist Circumference (cm)	-0.83 (-2.17, 0.52)	-0.35 (-1.72, 1.02)	-0.89 (-2.16, 0.38)
Systolic BP (mmHg)	-0.43 (-2.17, 1.32)	0.33 (-1.43, 2.09)	-0.71 (-2.34, 0.93)
Arterial distensibility (mm)	0.01 (-0.01, 0.03)	-0.00 (-0.02, 0.01)	0.01 (-0.01, 0.02)
cIMT(mm) *	0.00 (-0.01, 0.01)	0.00 (-0.01, 0.01)	00.01 (-0.00, 0.01)
CRP (% change) †	-21.76 (-37.74, -5.79)	-23.40 (-39.68, -7.11)	-18.50 (-33.63, -3.36)
HDL (mmol/L) ‡	0.05 (-0.00, 0.11)	0.03 (-0.03, 0.09)	0.05 (-0.01, 0.10)
Triglycerides (mmol/L)	-0.08 (-0.16, -0.00)	-0.05 (-0.13, 0.03)	-0.07 (-0.15, 0.00)
Insulin (% change)	-4.26 (-13.11, 4.59)	-2.56 (-12.30, 5.19)	-5.69 (-13.91, 2.54)
Proinsulin (% change)	-3.87 (-12.17, 4.44)	-0.77 (-8.91, 7.36)	-3.63 (-11.36, 4.11)

*cMIT - carotid intima-media thickness
†CRP- C- reactive protein
‡HDL- high density lipoprotein
Reference group=participants who never breastfed
All outcomes were adjusted for age at delivery, race, pre-pregnancy BMI, smoking, marital status, and gestational diabetes. Blood pressure outcomes were further adjusted for use of antihypertensive medications. Glucose, insulin, and proinsulin outcomes were further adjusted for diabetes medications. LDL-cholesterol and HDL-cholesterol outcomes were further adjusted for cholesterol lowering medications.

Table S30. Sensitivity analysis for associations of exclusive breastfeeding with cardiometabolic outcomes by hypertension during pregnancy restricted to multiparous women

	< 1 month	1-<3 months	3-<6 months
	Mean difference (95% CI)	Mean difference (95% CI)	Mean difference (95% CI)
No Hypertensive disorders of pregnancy			
Diastolic BP (mmHg)	-0.32 (-1.82, 1.18)	-1.09 (-2.61, 0.43)	-0.58 (-1.99, 0.84)
Mean arterial pressure	-0.36 (-1.80, 1.09)	-0.62 (-2.08, 0.85)	-0.62 (-1.98, 0.74)
LDL cholesterol (mmol/L)*	-0.07 (-0.20, 0.05)	0.04 (-0.08, 0.16)	-0.08 (-0.20, 0.04)
Glucose (% change)	-0.93 (-2.59 ,0.73)	0.60 (-1.03, 2.22)	-0.17 (-1.70, 1.37)
Hypertensive disorders of pregnancy			
Diastolic BP (mmHg)	-2.88 (-7.31, 1.55)	-4.68 (-9.49, 0.14)	-4.03 (-8.44, 0.38)
Mean arterial pressure	-2.67 (-6.76, 1.41)	-4.66 (-9.10, -0.21)	-4.73 (-8.80, -0.67)
LDL cholesterol (mmol/L)*	-0.04 (-0.33, 0.25)	-0.28 (-0.59, 0.02)	-0.22 (-0.50, 0.06)
Glucose (% change)	-0.80 (-5.60, 4.00)	-3.02 (-8.17, 2.14)	-3.73 (-8.54, 1.09)

*LDL- low density lipoprotein

Reference group=participants who never breastfed

All outcomes were adjusted for age at delivery, race, pre-pregnancy BMI, smoking, marital status, and gestational diabetes. Blood pressure outcomes were further adjusted for use of antihypertensive medications. Glucose, insulin, and proinsulin outcomes were further adjusted for diabetes medications. LDL-cholesterol and HDL-cholesterol outcomes were further adjusted for cholesterol lowering medications.
